# Mitochondrial genome of *Cordyceps blackwelliae*: organization, transcription, and evolutionary insights into *Cordyceps*

**DOI:** 10.1186/s43008-023-00118-5

**Published:** 2023-07-06

**Authors:** Yong-Jie Zhang, Xiang-Ping Fan, Jia-Ni Li, Shu Zhang

**Affiliations:** 1 School of Life Science, Shanxi University, Taiyuan, 030006 China; 2 Key Laboratory of Chemical Biology and Molecular Engineering of Ministry of Education, Shanxi University, Taiyuan, 030006 China; 3 Key Laboratory of Microbial Resources Collection and Preservation, Ministry of Agriculture and Rural Affairs, Beijing, 100081 China

**Keywords:** *Cordyceps blackwelliae*, Mitogenome, Transcriptome, Phylogeny, Cordycipitaceae, Evolution

## Abstract

*Cordyceps* is a diverse genus of insect pathogenic fungi, with about 180 accepted species, including some well-known ones used as ethnic medicine and/or functional food. Nevertheless, mitogenomes are only available for four members of the genus. The current study reports the mitogenome of *Cordyceps blackwelliae*, a newly described entomopathogenic fungus. The 42,257-bp mitogenome of the fungus encoded genes typically found in fungal mitogenomes, and a total of 14 introns inserted into seven genes, including *cob* (1 intron), *cox1* (4), *cox3* (3), *nad1* (1), *nad4* (1), *nad5* (1), and *rnl* (3). RNA-Seq analysis revealed differential expression of mitochondrial genes and supported annotations resulting from in silico analysis. There was clear evidence for polycistronic transcription and alternative splicing of mitochondrial genes. Comparison among mitogenomes of five different *Cordyceps* species (i.e., *C. blackwelliae*, *C. chanhua*, *C. militaris*, *C. pruinosa*, and *C. tenuipes*) revealed a high synteny, with mitogenome size expansion correlating with intron insertions. Different mitochondrial protein-coding genes showed variable degrees of genetic differentiation among these species, but they were all under purifying selection. Mitochondrial phylogeny based on either nucleotide or amino acid sequences confirmed the taxonomic position of *C. blackwelliae* in Cordycipitaceae, clustering together with *C. chanhua*. This study promotes our understanding of fungal evolution in *Cordyceps*.

**Supplementary Information:**

The online version contains supplementary material available at 10.1186/s43008-023-00118-5.

## INTRODUCTION

*Cordyceps*, the type genus of the family Cordycipitaceae (Hypocreales, Ascomycota), is the most diverse genus of insect pathogenic fungi, with about 180 accepted species (https://nmdc.cn/fungalnames/). They are parasitic on spiders and insects of various orders (e.g., Coleoptera, Diptera, Hemiptera, Hymenoptera, Lepidoptera, and Orthoptera) (Mongkolsamrit et al. 2020), showcasing important sources of treating various disorders due to the presence of multiple bioactive constituents (Olatunji et al. 2018). Discrimination of *Cordyceps* from closely related genera (e.g., *Blackwellomyces* and *Samsoniella*) and identification of different *Cordyceps* species mainly rely on molecular data (Mongkolsamrit et al. 2020). Traditionally, nuclear genes like *nrSSU*, *nrLSU*, *tef1*, *rpb1*, and *rpb2* are frequently used in molecular phylogeny of *Cordyceps* and related fungal taxa (Kepler et al. 2017; Sung et al. 2007).

Mitochondrion is a functionally important organelle that is almost universally present in eukaryotic cells and contains its own genetic material (Sandor et al. 2018; Zhang 2022). The genetic material in mitochondria is called mitochondrial DNA (mtDNA) or mitochondrial genome (mitogenome). MtDNA has a number of special biological properties, such as its multiple copy nature within an individual cell, dominantly uniparental inheritance, evolution in a nearly neutral fashion, and clock-like nature of its substitution rate (Galtier et al. 2009; Zhang 2022). These traits make mtDNA a molecular marker widely used in eukaryotic ecology, phylogeny, and population genetics (Corradi and Bonen 2012; Galtier et al. 2009). Because of its popularity, mitogenome data from a growing number of eukaryotic organisms have been available over recent years. For fungi, there were 334 different species with available mitogenomes as of late June, 2019 (Zhang 2022). Only four members of *Cordyceps*, however, have available mitogenomes as of January 2023, including *C. chanhua* (Fan et al. 2019), *C. militaris* (Sung 2015), *C. pruinosa* (GenBank: MN515031), and *C. tenuipes* (Li et al. 2019).

Different fungal mitogenomes show variations in a variety of aspects, such as gene organization, gene and intron content (Zhang 2022). Comparing mitogenomes of different species is helpful to reveal their evolutionary relationships. Such comparisons have been performed for many fungal lineages, such as Cordycipitaceae (Fan et al. 2019), Ophiocordycipitaceae (Zhang et al. 2017c), Hypocreales (Ren et al. 2021), and Mucoromycota (Zhang et al. 2022), using mitogenome data available at that time. Comprehensive comparison of mitogenomes among different *Cordyceps* species, however, has not been performed.

Although mitogenome sequences of a growing number of fungi are reported, only few papers regarding fungal mitogenomes investigated expression of mitochondrial genes. Expression quantification of mitochondrial genes was only performed for several yeasts, including *Candida albicans* (Kolondra et al. 2015), *Saccharomyces cerevisiae* (Turk et al. 2013), and *Schizosaccharomyces pombe* (Shang et al. 2018), and filamentous fungi, including *Hirsutella thompsonii* (Wang et al. 2018), *Ophiocordyceps sinensis* (Li et al. 2015), *Pestalotiopsis fici* (Zhang et al. 2017a), and *Tolypocladium inflatum* (Zhang et al. 2017b). Differential expression of mitochondrial genes was always demonstrated in these studies. Mitochondrial genes in *Candida albicans*, *Saccharomyces cerevisiae*, and *Schizosaccharomyces pombe* were further found to be transcribed into 8 (Kolondra et al. 2015), 11 (Turk et al. 2013), and two (Schäfer et al. 2005) primary polycistronic units, respectively. Evidence of alternative splicing of intron-containing genes was clear in *Saccharomyces cerevisiae* (Turk et al. 2013). As far as we know, it is still unclear if a filamentous fungus transcribes mitochondrial genes as polycistronic transcription units and if there is alternative splicing for mitochondrial genes in filamentous fungi.

*Cordyceps blackwelliae* is an entomopathogenic fungus within the family Cordycipitaceae (Hypocreales, Ascomycota). It was firstly described as a novel species from Thailand in 2018, infecting lepidopteran/coleopteran larva/pupa in leaf litter or underside of leaves (Mongkolsamrit et al. 2018). Since then, the fungus has been known from Vietnam and China, seemingly constricting to tropical and subtropical regions (Fan et al. 2022). The fungus is easy to grow fruiting bodies in artificial environment and displays antioxidant, antimicrobial, and fibrinolytic activities (Fan et al. 2022). Obviously, the fungus represents a valuable resource of great development potential. Genome-level studies of the fungus will help to understand its evolution and phylogenetic relationship with other *Cordyceps* species.

Since species of the same genus share a most recent common ancestor, we hypothesize that different *Cordyceps* species are conserved in their mitogenome organization. Alternative splicing should be existent for intron-containing mitochondrial genes in filamentous fungi. To test the two hypotheses, we assembled the mitogenome of *C. blackwelliae*, investigated expression of its mitochondrial genes, and compared mitogenomes of different *Cordyceps* species. Our specific goals are (1) to disclose the mitogenome organization in *C. blackwelliae*, (2) to examine mitochondrial transcription pattern of the fungus, and (3) to gain evolutionary insights of *Cordyceps* by phylogenetics and comparative mitogenomics. This study will enrich the available mitogenome information in *Cordyceps* and provide valuable reference for understanding fungal evolution in *Cordyceps*.

## MATERIALS AND METHODS

### Sample information

*Cordyceps blackwelliae* strain ZYJ0835 was isolated from a fresh cordyceps specimen collected from Danxia Mountain, Shaoguan, Guangdong, China in June 6, 2019. It was identified based on both morphology and multi-gene phylogeny (Fan et al. 2022). The strain was deposited in China Center for Type Culture Collection (CCTCC) under No. M2022663. The strain was cultivated on potato dextrose agar (PDA) medium covered with a piece of cellophane paper at 25 °C for 7 days. Mycelia were collected and frozen quickly in liquid nitrogen to be used for DNA and RNA extraction.

### Mitogenome assembly and annotation

The extracted total DNA was randomly sheared to fragments of ~ 350 bp, followed by sequencing on an Illumina NovaSeq platform in 2 × 150 bp reads at Novogene Co. Ltd. (Tianjin, China). The original sequencing data were filtered for quality. During the data processing steps, paired reads were discarded if more than 10% of bases were uncertain in either one read, or if the proportion of low quality bases (Phred quality ≤ 20) was over 40% in either one read. Mitogenome sequences were de novo assembled using filtered data with two independent programs NOVOPlasty v4.3.1 (Dierckxsens et al. 2017) and GetOrganelle v1.7.5 (Jin et al. 2020). The mitogenome sequences were mainly annotated using MFannot (https://megasun.bch.umontreal.ca/apps/mfannot/) based on the mold mitochondrial genetic code (i.e., genetic code 4), but with necessary manual correction. Detailed annotation methods referred to those described previously (Ren et al. 2021; Zhang et al. 2017b). Open reading frames (ORFs) within introns and intergenic regions were identified using ORF Finder (https://www.ncbi.nlm.nih.gov/orffinder/), and only ORFs ≥ 300 bp were considered. The AUG codon was preferred as the start codon of all protein-coding genes (PCGs), but alternative initiation codons were also acknowledged when there was no available AUG start codon. Introns in rRNA and PCGs were named after their insertion sites according to the established nomenclatures (Johansen and Haugen 2001; Zhang and Zhang 2019a). The secondary structure of tRNAs was analyzed using tRNAscan-SE (http://lowelab.ucsc.edu/tRNAscan-SE/). The circular map of the mitogenome was visualized using OGDRAW (Greiner et al. 2019).

### Transcriptome sequencing

To understand the transcription of mitochondrial genes and to verify our above annotations of mitochondrial genes, RNA-Seq was performed using mycelia cultivated on PDA plates. The extracted total RNA was treated with two different methods. In method 1, mRNA was enriched from total RNA using polyT oligo-attached magnetic beads (i.e., polyA RNA capture strategy). In method 2, rRNA was removed from total RNA using probes (i.e., rRNA depletion strategy). For RNA samples resulting from each strategy, fragmentation was carried out using divalent cations under elevated temperature in NEB Fragmentation Buffer. Fragments of 350– 400 bp were extracted and used for sequencing library construction using NEBNext® Ultra™ RNA Library Prep Kit for Illumina®. Sequencing was then performed on an Illumina NovaSeq platform in 2 × 150 bp reads at Novogene Co. Ltd. (Tianjin, China). Original reads were filtered to discard paired reads if there were uncertain bases in either one read, or if the proportion of low quality bases (Phred quality ≤ 20) was over 50% in either one read. Filtered reads were mapped to the *C. blackwelliae* mitogenome using STAR v2.7.10b (Dobin et al. 2013). Expression of mitochondrial genes was quantified using RSEM v1.3.1 (Li and Dewey 2011). FPKM values (fragments per kilobase exon model per million mapped reads) were calculated, and genes were considered to be expressed if FPKM > 1.0. Visualization of sequencing depth for genes of interest was realized using IGV v2.15.2 (Thorvaldsdottir et al. 2013). Transcript assembly was performed using Trinity v2.14.0 (Grabherr et al. 2011), under the genome-guided mode or de novo mode. Comparison between the mitogenome and transcripts was performed using Easyfig v2.2.5 (Sullivan et al. 2011).

### Phylogenetic analysis of Hypocreales species

To determine the phylogenetic position of *C. blackwelliae* in Hypocreales, two datasets were used for phylogenetic analyses. One was the concatenated nucleotide sequences of 14 core PCGs (including *atp6*, *8–9*, *cob*, *cox1-3*, *nad1-6*, and *nad4L*), and the other was the concatenated amino acid sequences of these core PCGs. A total of 37 fungal taxa were employed as ingroups, including all species of Cordycipitaceae and representative species of other families in Hypocreales with available mitogenomes when this study was performed (Additional file 1: Table S1). Two species in Glomerellales, *Colletotrichum aenigma* and *Colletotrichum gloeosporioides*, were used as outgroups. Phylogenetic relationships were estimated using both Maximum likelihood (ML) and Bayesian (BI) approaches, with settings described in our previous publication (Ren et al. 2021). In interpreting phylogenetic confidence, we considered nodes strongly supported if they received posterior probability ≥ 0.95 (for BI) or bootstrap values ≥ 70% (for ML).

### Comparison among mitogenomes of five *Cordyceps species*

Besides *C. blackwelliae*, four other *Cordyceps* species have available mitogenomes when this study is performed. To have a basic understanding of mitogenome evolution in *Cordyceps*, we compared these five *Cordyceps* mitogenomes with respect to genome size, gene content, gene order, intron insertion, genetic distance, and selection pressure. Codon alignment were performed for PCGs shared by all mitogenomes using MEGA v11 (Tamura et al. 2021). The overall mean genetic distances of these PCGs among the five *Cordyceps* species were determined according to Kimura-2-parameter (K2P) substitution model using MEGA v11 (Tamura et al. 2021). The nonsynonymous (Ka) and synonymous (Ks) substitution rates were calculated using KaKs_Calculator v2.0 (Wang et al. 2010), with the following settings: genetic code Table 4 (mold mitochondrial code) and the YN method. The calculated Ka/Ks ratios were used to infer potential selection pressure on each gene, with a value greater than, equal to, or less than one indicating positive (diversifying) selection, neutral evolution, or purifying (negative) selection, respectively. All mitogenomes were adjusted to start from *rnl* and then aligned using Mauve (Darling et al. 2010) to visualize syntenies and to identify possible gene rearrangement events.

### Availability of data

The mitogenome sequence of *C. blackwelliae* was deposited to GenBank under the accession number OM403992. RNA-Seq data were submitted to the NCBI Sequence Read Archive (SRA) database under the BioProject accession number PRJNA912387.

## RESULTS

### Organization of the *C. blackwelliae* mitogenome

The two mitogenome assembly programs, NOVOPlasty and GetOrganelle, generated complete and identical mitogenome sequences, i.e., a circular molecule of 42,257 bp in length. Mitochondrial reads accounted for 2.1% of the overall reads. Different regions of the mitogenome showed certain variations on their sequencing depth; however, even the weakly-sequenced mtDNA regions had a sequencing depth higher than nuclear genes (Additional file 2: Fig. S1). On average, the sequencing depth of the mitogenome (~ 1006 ×) was 17-fold larger than that of the nuclear genome (~ 57 ×). No introgression of mitochondrial genes in the nuclear genome was revealed by local BLAST analysis.

**Fig. 1:**
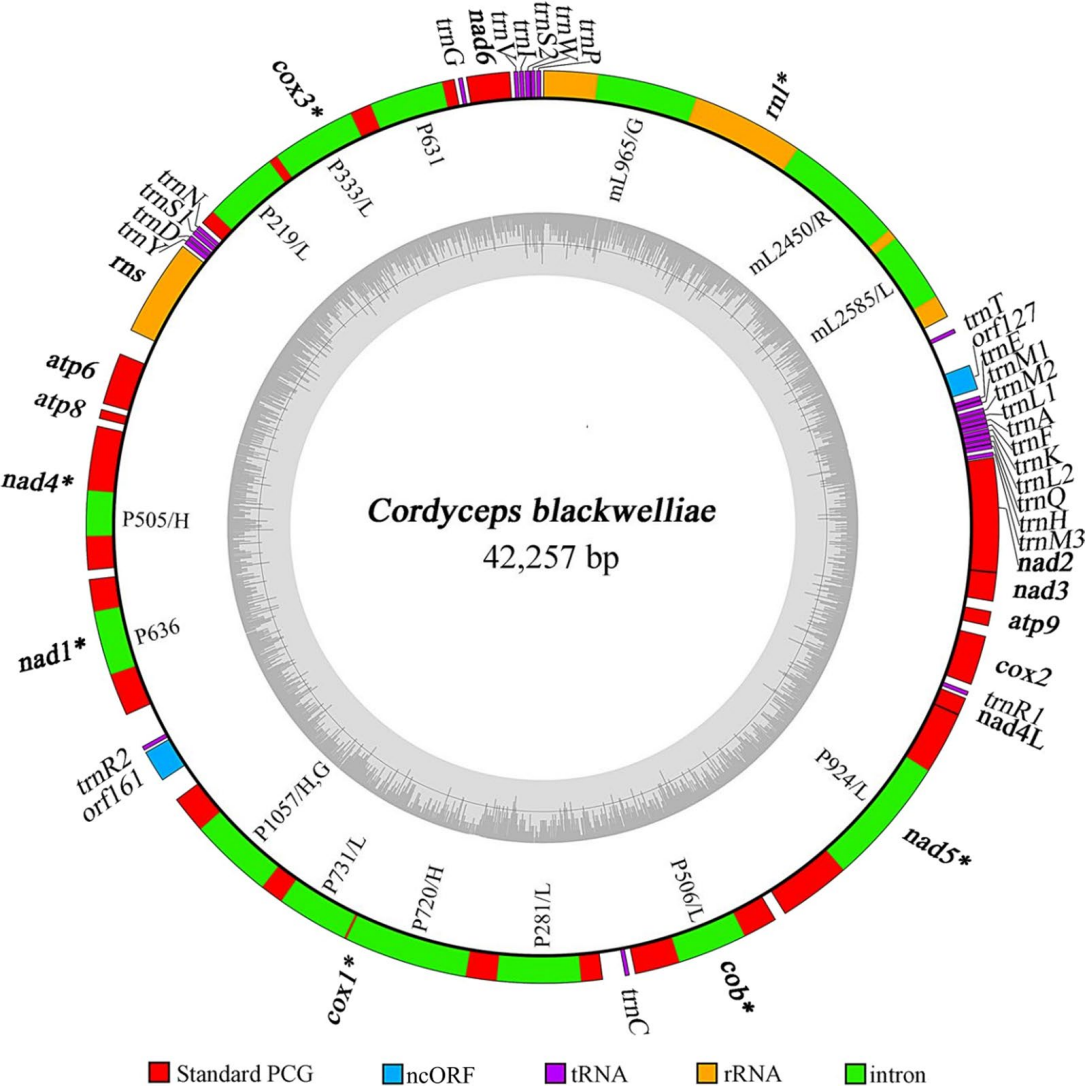
Circular map of the *C. blackwelliae* mitogenome. The outer ring marks relative positions of different genes, and the inner ring represents GC contents. All genes are transcribed at the same strand. Different kinds of genes/sequences are shown in different colours as indicated at the bottom of the figure. The 14 core PCGs and the two rRNA genes typically found in fungal mitogenomes are shown in bold. Intron-containing genes are marked by asterisks after their names. For introns, standard intron names (containing insertion point information) and intron-encoded proteins (G, GIY-YIG endonuclease; L, LAGLIDADG endonuclease; R, ribosomal protein; H, hypothetical protein) are given in the inner side of the outer ring. Please note that two introns, nad1P636 and cox3P631, do not contain an intronic ORF, and cox1P1057 contains two intronic ORFs. Refer to Tables 1 and 2 for details of the mitogenome annotations

There were 43 free-standing genes in the mitogenome, including two rRNA genes (*rnl* & *rns*), 25 tRNA genes, 14 typical PCGs coding for proteins of the oxidative phosphorylation system, and two intergenic ORFs (Fig. 1; Table 1). All genes were transcribed at the forward strand. As reported in most other fungal mitogenomes, the 14 typical PCGs encoded seven subunits of NADH dehydrogenase (*nad1–6*, *4L*), three subunits of cytochrome c oxidase (*cox1–3*), apocytochrome b (*cob*), and three subunits of ATP synthase (*atp6*,* 8*, *9*). The two intergenic ORFs (*orf127A* and *orf161*) encoded a LAGLIDADG endonuclease and a hypothetical protein, respectively. Unexpectedly, online BLASTx analysis of *orf127A* and its flanking sequences suggested that *orf127A* may originally encode an even longer LAGLIDADG endonuclease since stop codon mutations and frame shifts were detected in the flanking sequences of *orf127A*. We only identified an alternative initiation codon AUC for *orf127A* and failed to identify the standard initiation codon AUG.

**Table 1:** Gene features in the mitogenome of *Cordyceps blackwelliae*

Gene	Start	End	Length (bp)	Start codon	Stop codon	Anticodon	Note †
*rnl*	11	7344	7334				3 introns
*trnT*	7540	7610	71			UGU	
*orf127A*	8121	8504	384	AUC	UAA		L
*trnE*	8598	8670	73			UUC	
*trnM1*	8672	8742	71			CAU	
*trnM2*	8784	8856	73			CAU	
*trnL1*	8860	8941	82			UAA	
*trnA*	8945	9016	72			UGC	
*trnF*	9018	9090	73			GAA	
*trnK*	9097	9169	73			UUU	
*trnL2*	9171	9254	84			UAG	
*trnQ*	9258	9330	73			UUG	
*trnH*	9332	9405	74			GUG	
*trnM3*	9450	9521	72			CAU	
*nad2*	9529	11,238	1710	AUG	UAA		
*nad3*	11,238	11,669	432	AUG	UAA		
*atp9*	11,814	12,038	225	AUG	UAA		
*cox2*	12,189	12,938	750	AUG	UAA		
*trnR1*	13,041	13,111	71			GCG	
*nad4L*	13,149	13,418	270	AUG	UAA		
*nad5*	13,418	17,365	3948	AUG	UAA		1 intron
*cob*	17,523	19,756	2234	AUG	UAA		1 intron
*trnC*	19,837	19,908	72			GCA	
*cox1*	20,234	27,391	7158	AUG	UAA		4 introns
*orf161*	27,736	28,221	486	AUG	UAG		H
*trnR2*	28,251	28,321	71			UCU	
*nad1*	28,837	30,921	2085	AUG	UAA		1 intron
*nad4*	31,052	33,199	2148	AUG	UAA		1 intron
*atp8*	33,306	33,452	147	AUG	UAA		
*atp6*	33,533	34,315	783	AUG	UAA		
*rns*	34,691	36,156	1466				
*trnY*	36,197	36,281	85			GUA	
*trnD*	36,285	36,357	73			GUC	
*trnS1*	36,380	36,460	81			GCU	
*trnN*	36,469	36,539	71			GUU	
*cox3*	36,598	40,952	4355	AUG	UAA		3 introns
*trnG*	41,004	41,074	71			UCC	
*nad6*	41,124	41,777	654	AUG	UAA		
*trnV*	41,829	41,900	72			UAC	
*trnI*	41,910	41,981	72			GAU	
*trnS2*	41,993	42,076	84			UGA	
*trnW*	42,078	42,149	72			UCA	
*trnP*	42,164	42,235	72			UGG	

^†^For intron-containing genes, the number of introns present in corresponding genes is indicated in the last column. For non-conserved ORFs, proteins encoded by them are indicated (L, LAGLIDADG endonuclease; H, hypothetical protein)

**Fig. 2:**
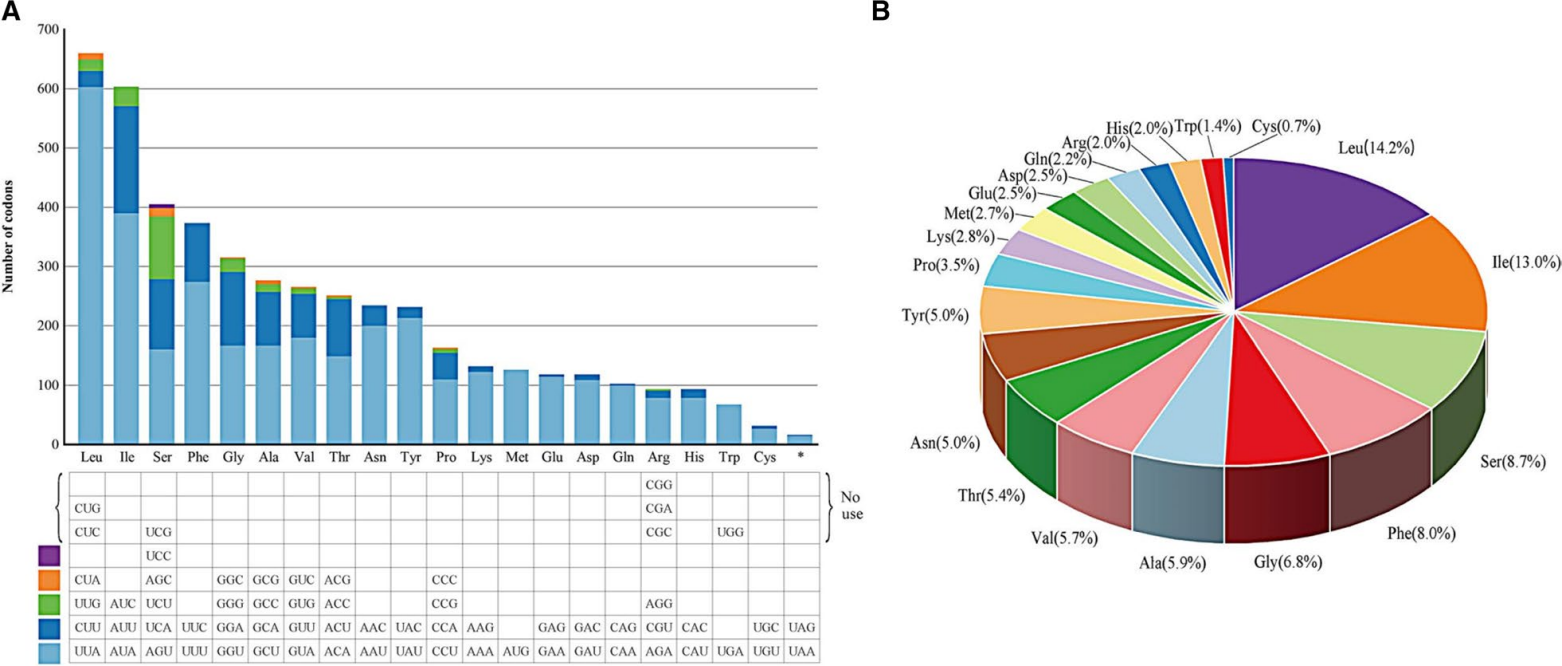
Codon usage (**A**) and amino acid frequency (**B**) for 16 free-standing PCGs in the *C. blackwelliae* mitogenome. The 16 PCGs include 14 typical PCGs and two intergenic ORFs. Please note that several codons are never used by these PCGs, and the UGA codon is used multiple times to code for tryptophan

As expected, the mitogenome contained higher abundance of bases A (37.9%) & T (36.3%) than G (14.5%) & C (11.3%). The high AT content (74.2%) was also reflected in codon usage of the 16 PCGs (14 typical PCGs plus two intergenic ORFs) (Fig. 2A). Those most-frequently-used codons were exclusively composed of bases A & U, such as UUA(L) (603 times), AUA(I) (390), UUU(F) (274), UAU(Y) (213), and AAU(N) (200). Several codons rich in G & C (i.e., CUG, CUC, UCG, CGG, CGA, CGC, and UGG) were never used. It is noteworthy that codon UGA (i.e., the stop codon in standard genetic code) occurred a total of 67 times in 12 out of the 16 PCGs (absent from *atp8*, *atp9*, *nad3*, and *nad4L*), supporting that *C. blackwelliae* employed the mold mitochondrial genetic code (i.e., Code 4) to translate its mitochondrial PCGs. Amino acids Leu, Ile, Ser, Phe, and Gly were more abundant than others, and they accounted for half (50.6%) of the overall amino acids in these PCGs (Fig. 2B). All amino acids preferred to use AT-rich codons when applicable (Fig. 2A). The high AT content of the mitogenome may be the reason why mtDNA is prone to mutation.

The 25 tRNAs translated the occurring codons into all 20 standard amino acids (Table 1). There were three tRNAs for methionine all with the CAU anticodons and two tRNA versions for arginine, leucine, and serine with the usual anticodon differences. These *trn* genes mostly clustered at the *rnl*/*nad2* intergenic region (12 in number), followed by the *nad6*/*rnl* (5) and *rns*/*cox3* (4) intergenic regions. The remaining four *trn* genes scattered at *cox2/nad4L*, *cob/cox1*, *cox1/nad1*, and *cox3/nad6* intergenic regions (Fig. 1; Table 1). All tRNAs folded into classical cloverleaf structures, with five (i.e., trnL1, trnL2, trnS1, trnS2, and trnY) having extra loops (Additional file 2: Fig. S2). These five tRNAs with extra loops (81–85 nt) were larger than other tRNAs (71–74 nt). G-U mismatch occurred 41 times in all tRNAs except trnN, trnR2, and trnY (Additional file 2: Fig. S2).

The mitogenome is rather compact. Specifically, two gene pairs *nad2*/*nad3* and *nad4L*/*nad5* had special organization where the latter genes in each gene pair overlapped by one base with the former genes. Other physically neighboring genes were separated by at least one base (Table 1). This 1-bp interval occurred between several *trn* genes, such as *trnE/trnM1*, *trnA/trnF*, and *trnS2/trnW*. The two longest intervals located between *trnR* and *nad1* (515 bp) and between *trnT* and *orf127A* (510 bp). Overall, intergenic regions reached a total length of 3,830 bp, accounting for 9.1% of the mitogenome.

### Introns and intronic ORFs in the *C. blackwelliae* mitogenome

There were a total of 14 introns in the mitogenome (Fig. 1, Table 2). These introns invaded into seven different genes, including *cob* (1 intron), *cox1* (4), *cox3* (3), *nad1* (1), *nad4* (1), *nad5* (1), and *rnl* (3). These introns all belonged to the group I intron family and fell into five specific subgroups, namely IA (2 introns), IB (8), IC1 (1), IC2 (2), and derived I (1). These group I introns were characterized by preceding exons ending with “T” and introns ending with “G”, with the exception of cox1P281 whose upstream exon ended with “C” instead of “T”. The overall length of intronic regions (including intronic ORFs) was 18,153 bp, accounting for 43.0% of the mitogenome. This indicates that introns contribute greatly to mitogenome expansion.

**Table 2:** Features of introns and intronic ORFs characterized in the *C. blackwelliae* mitogenome

Intron/intronic ORF	Start	End	Length (bp)	Type	Standard name	Start codon	Stop codon	IEP†
*rnl*-i1	815	2327	1513	IC1	mL965			
*orf103*	1594	1905	312			AUU	UAG	G
*rnl*-i2	3982	5805	1824	IA	mL2450			
*orf456*	4270	5640	1371			AUG	UAA	R
*rnl*-i3	5943	7005	1063	IA	mL2585			
*orf102*	6227	6535	309			UUA	UAA	L
*nad5*-i1	14,342	16,294	1953	IB	nad5P924			
*orf386*	14,342	15,502	1161			ND	UAA	L
*cob*-i1	18,029	19,089	1061	IB	cobP506			
*orf319*	18,030	18,989	960			ND	UAA	L
*cox1*-i1	20,548	21,818	1271	IB	cox1P281			
*orf301*	20,549	21,454	906			ND	UAA	L
*cox1*-i2	22,258	24,129	1872	IB	cox1P720			
*orf535*	22,485	24,092	1608			AUG	UAA	H
*cox1*-i3	24,141	25,239	1099	IB (3')	cox1P731			
*orf336*	24,148	25,158	1011			AUA	UAA	L
*cox1*-i4	25,566	26,837	1272	IB	cox1P1057			
*orf129*	25,568	25,957	390			AUA	UAA	H
*orf112*	26,490	26,828	339			AUG	UAA	G
*nad1*-i1	29,467	30,447	981	IB	nad1P636			
*nad4*-i1	31,548	32,246	699	IC2	nad4P505			
*orf127B*	31,550	31,933	384			ND	UAA	H
*cox3*-i1	36,817	37,951	1135	IB	cox3P219			
*orf308*	36,817	37,743	927			ND	UAA	L
*cox3*-i2	38,066	39,347	1282	IC2	cox3P333			
*orf419*	38,066	39,325	1260			ND	UAA	L
*cox3*-i3	39,646	40,773	1128	I*	cox3P631			

^†^IEP, intron encoded protein. G, GIY-YIG endonuclease; L, LAGLIDADG endonuclease; R, ribosomal protein; H, hypothetical protein

All introns except two (i.e., nad1P636 and cox3P631) contained putative ORFs encoding for ribosomal protein S3 (in mL2450), LAGLIDADG endonucleases (in mL2585, nad5P924, cobP506, cox1P281, cox1P731, cox3P219, and cox3P333), GIY-YIG endonucleases (in mL965 and cox1P1057), or hypothetical proteins (in cox1P720, cox1P1057, and nad4P505) (Fig. 1, Table 2). Two intronic ORFs (encoding GIY-YIG endonuclease or hypothetical protein) were simultaneously identified in cox1P1057. The two ORF-lacking introns (nad1P636 and cox3P631) seemed to be remnants of intron degeneration. In addition, four other introns (i.e., mL965, mL2585, cox1P1057, and nad4P505) are likely degenerating due to stop codon mutations and/or frame shifts.

### Transcription of mitochondrial genes in *C. blackwelliae*

**Fig. 3:**
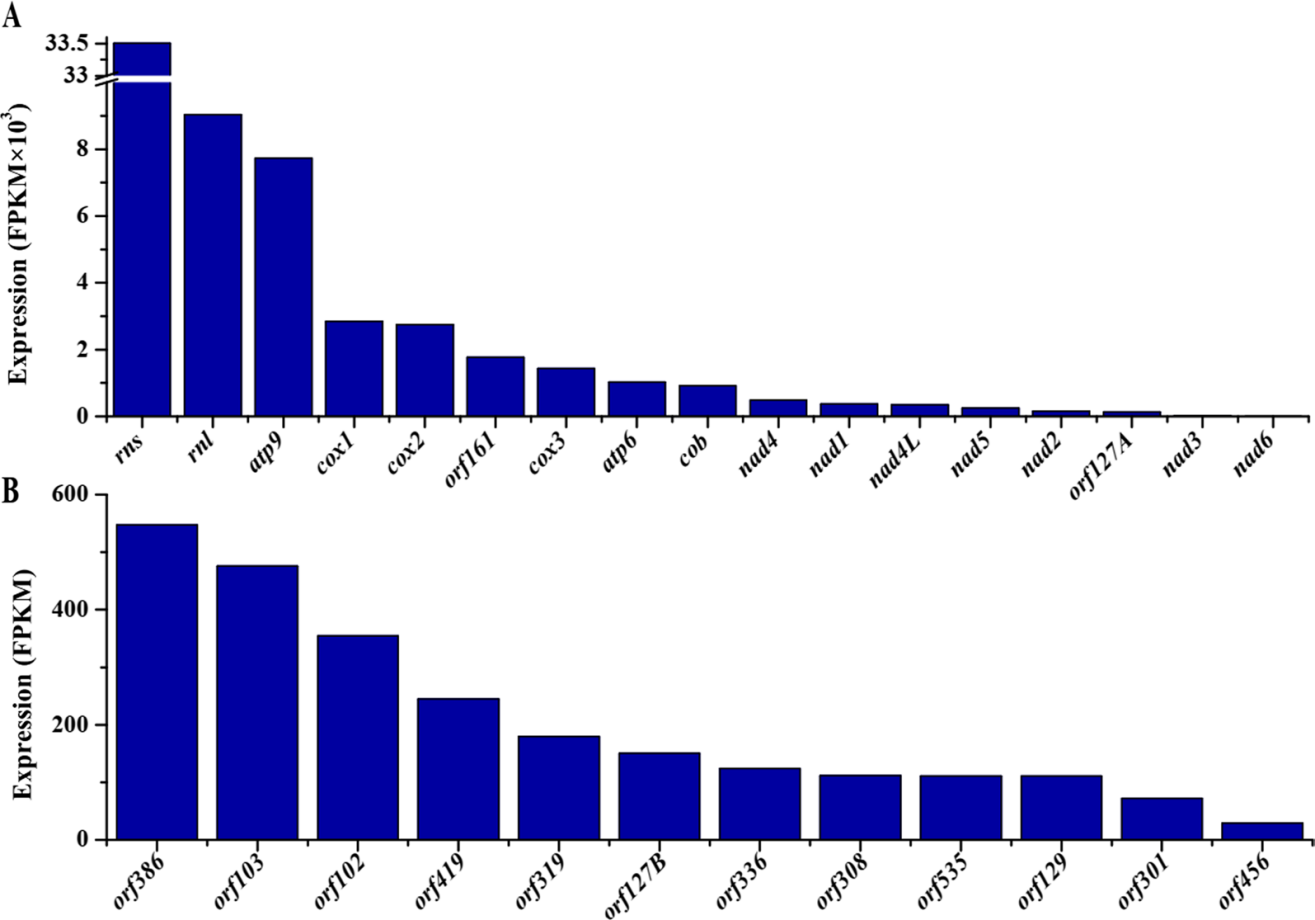
Expression of free-standing genes (**A**) and intronic ORFs (**B**) in the *C. blackwelliae* mitogenome. Please refer to Additional file 1: Table S2 for details of the FPKM values. Please note that the free-standing genes *atp8* and all *trn* genes, and the intronic ORF *orf112* have a FPKM value of zero, and thus they are not shown in the figure

Expression of mitochondrial genes was validated by RNA-Seq analyses, where RNA samples were treated with two different strategies, namely polyA RNA capture (PA) and rRNA depletion (RD). Of the initial sequencing reads generated by the two different strategies, 65,069,630 (for PA) or 72,767,668 (for RD) reads passed through quality filtration. Among these filtered reads, 350 (< 0.001%) and 7,206,016 (9.91%) were associated with mitochondrial genes in the PA and RD strategies, respectively. This was consistent with the direct observation of reads mapping figure (Additional file 2: Fig. S3). According to FPKM values, just weak expression of few genes (e.g., *rns*, *rnl*, *cox2*) was detected in the PA strategy, whereas strong expression of most genes was detected in the RD strategy (Additional file 1: Table S2). In the RD strategy, the FPKM values of *atp8*, the intronic *orf112*, and all *trn* genes were zero. However, reads mapping to these genes were detected though at a relatively low sequencing depth (Additional file 2: Fig. S4). All other genes were highly expressed but with clear variation on their expression levels (Fig. 3; Additional file 1: Table S2). For free-standing genes, the two rRNA genes (*rns* & *rnl*) displayed higher FPKM values than PCGs. Different PCGs showed 3654-fold differential expression between *atp9* with the highest FPKM value and *nad6* with the lowest FPKM value. For intronic ORFs, *orf386* (in nad5P924) and *orf456* (i.e., *rps3* in mL2450) had the maximum and minimum FPKM values, respectively (18-fold difference). Some intronic ORFs (e.g., *orf386*) even showed higher FPKM values than free-standing PCGs (e.g., *nad4*).

Since the number of mitochondrial reads in the RD strategy was more abundant than that in the PA strategy, only reads from the RD strategy were employed in subsequent analyses. Mitochondrial reads from the RD strategy were assembled using the genome-guided mode of Trinity. This generated 32 transcript contigs, whose lengths ranged from 365 to 13,222 nt, with a N50 of 10,890 nt and a total length of 107,116 nt. Local BLASTN searches of mitochondrial genes (using exon sequences as queries) against the assembled transcripts revealed that all free-standing (including two rRNA genes, 14 core PCGs, 25 tRNA genes, and two intergenic ORFs; 43 in total) and intronic (including 13 intronic ORFs) genes transcribed actively (Additional file 1: Table S3). This was also true for those genes with a zero FPKM value (i.e., *atp8*, the intronic *orf112*, and all *trn* genes); that is, transcript contigs containing these genes were present.

Local BLASTN searches of the complete mitogenome against the assembled transcripts revealed that transcription of mitochondrial genes could be represented or covered by four transcripts (Fig. 4). PCR assays for gaps between the four transcripts further showed that adjacent transcripts may be connected (Additional file 2: Fig. S5). This suggests that adjacent mitochondrial genes (plus intergenic regions) transcribe together, and the mitogenome may transcribe into one long polycistronic transcript.

**Fig. ﻿4:**
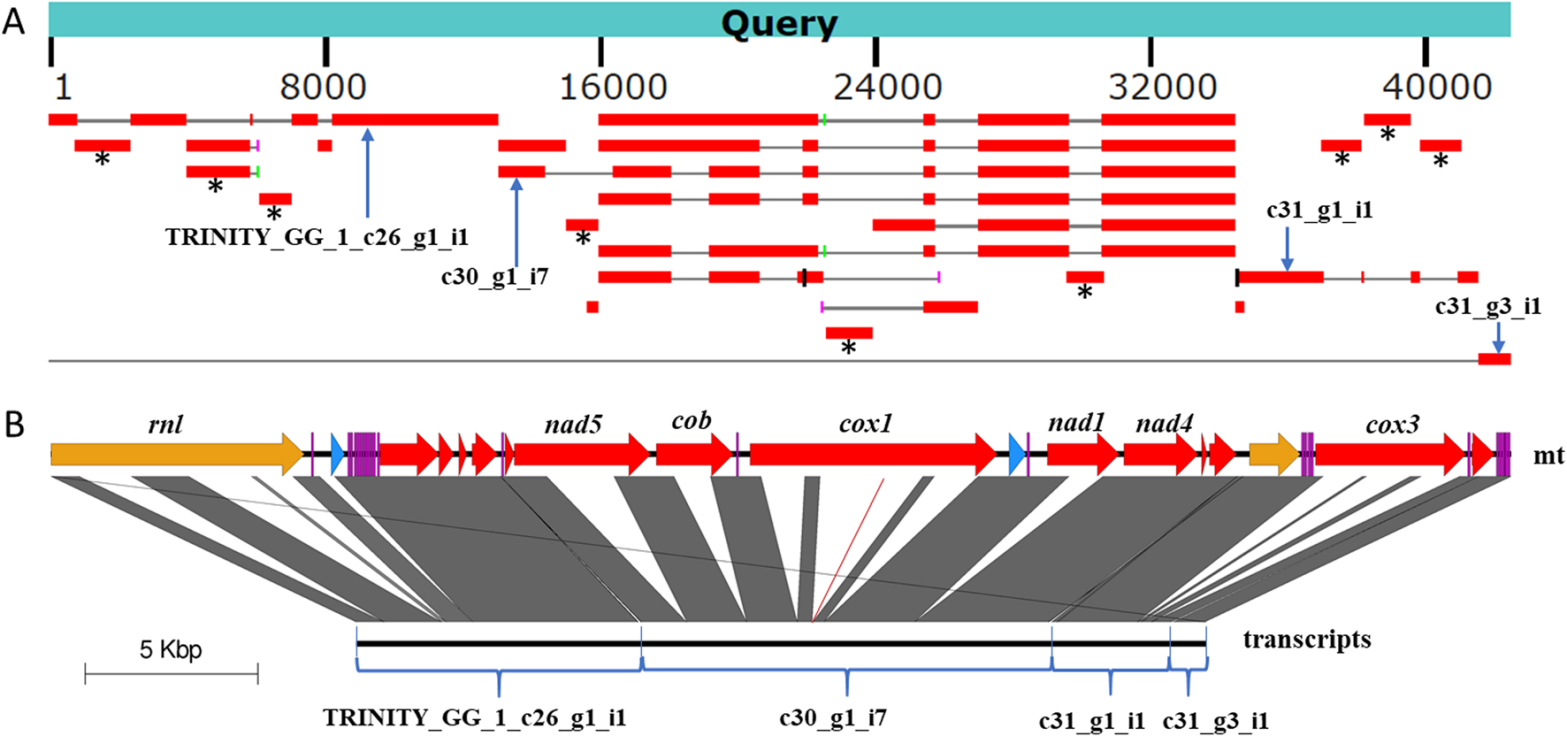
Comparison between the *C. blackwelliae* mitogenome and the assembled transcripts. (**A**) BLASTN analysis of the mitogenome against the 34 transcripts assembled under the genome-guided mode of Trinity. Four transcripts representing transcription of almost all mitochondrial genes are indicated by arrows. Several independent transcripts corresponding to introns are indicated by black asterisks. (**B**) Comparison between the mitogenome (mt) and the four transcripts marked by arrows in Panel A. In the mitogenome map, different kinds of genes are colored using the same coloring schemes as Fig. 1. Only the seven intron-containing genes are marked, and other genes can be known by referring to Fig. 1 or Table 1. The four transcripts are manually connected with 10 N bases (for convenience of drawing the figure), and their actual connections are later verified by PCR assays as shown in Additional file 2: Fig. S5. Please also note that the 3^rd^ exon of *cox1* is extremely short (11 bp in length). Such a short exon cannot be shown by the genome comparison visualizer Easyfig, and hence we artificially add a brown line to indicate the position of the exon. By this way, all introns except nad4P505 are seen to be excised

Local BLASTN searches against the assembled transcripts also supported our above annotations for all intron-containing genes with the exception of *nad4* (Additional file 1: Table S4). Although local BLASTN searches failed to support the splicing of the *nad4* intron (nad4P505), RT-PCR confirmed the excision of the *nad4* intron (Additional file 2: Fig. S5). The splicing of the *nad4* intron was also supported by local BLASTN searches against de novo assembled transcripts (Additional file 2: Fig. S6).

**Fig. 5:**
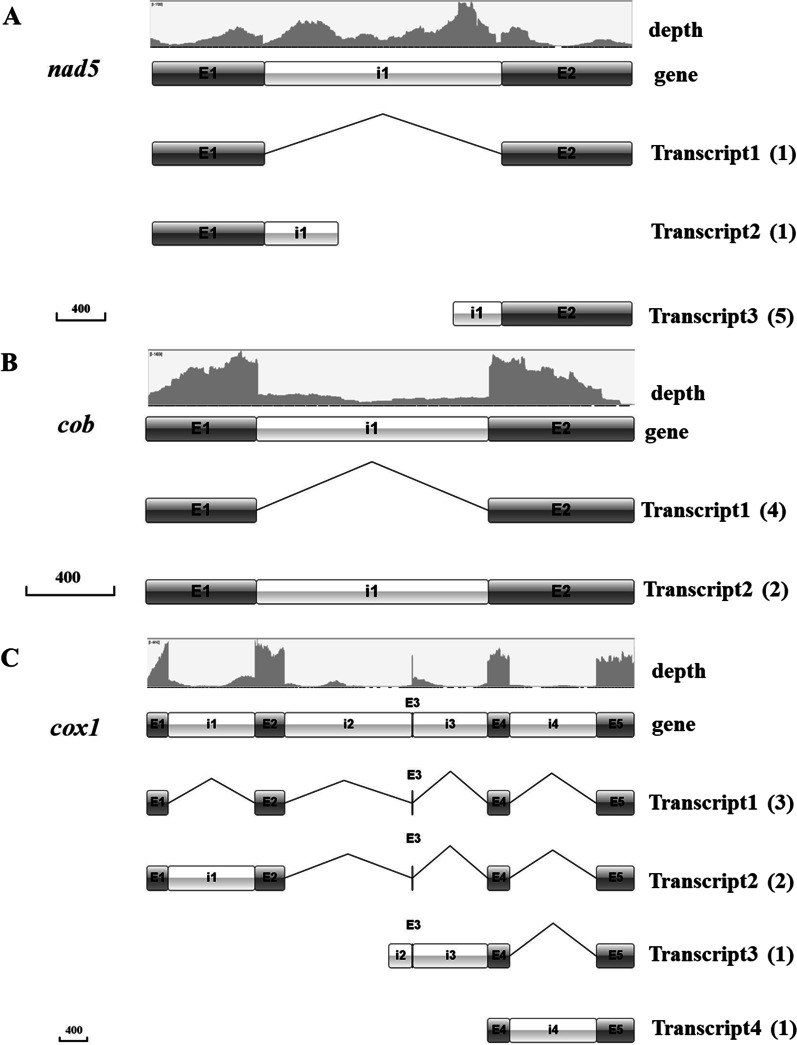
Alternative splicing of mitochondrial genes in *C. blackwelliae*. Three genes were found to have alternative splicing, namely *nad5* (**A**), *cob* (**B**), and *cox1* (**C**). In each panel, the sequencing depth of reads, gene structure, and all transcripts detected in RNA-Seq data are shown

Evidence for alternative splicing (mainly intron retention event) was clear for three intron-containing genes (i.e., *cob*, *cox1*, and *nad5*), with each having more than two different transcription isoforms (Fig. 5; Additional file 1: Table S4). Specifically, for *cob*, we identified transcripts with its intron either deleted (4 transcripts) or retained (2 transcripts). For *nad5*, we identified one transcript lacking its intron, one transcript consisting of the first exon and partial intron, and five transcripts consisting of partial intron and the second exon. For *cox1*, we identified three transcripts with all four introns deleted, two transcripts containing the first intron (cox1P281) and deleting other introns, one transcript containing partial or full sequence of the second (cox1P720) and third (cox1P731) introns, and one transcript containing the fourth intron (cox1P1057).

### Phylogenetic analyses based on mitochondrial sequences

**Fig. 6:**
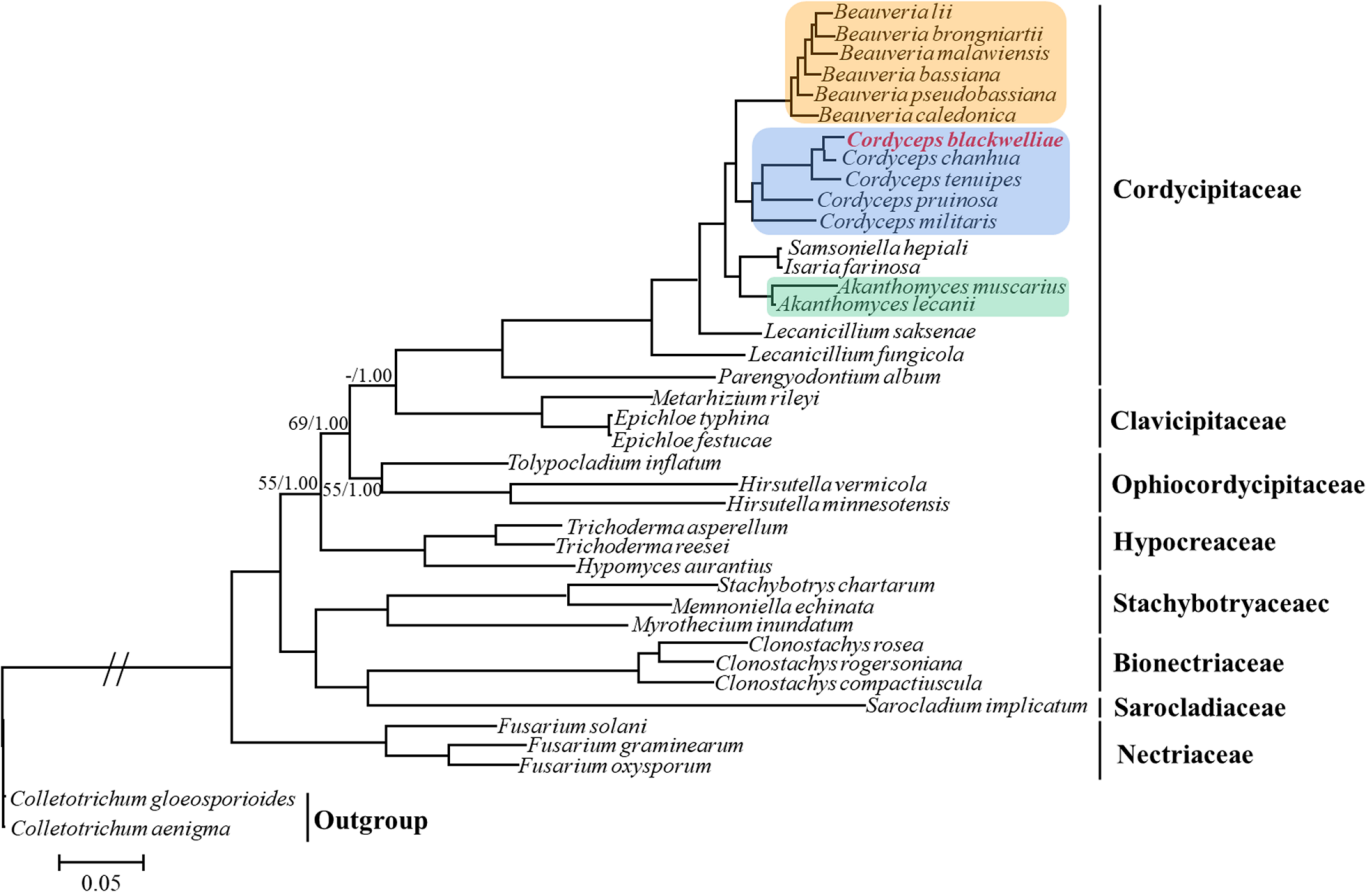
Phylogenetic analysis of Hypocreales species based on concatenated nucleotide sequences of 14 typical mitochondrial PCGs. The tree shown here is the single best topology recovered from ML. Support values (ML/BI) are indicated for nodes if they receive posterior probability < 0.95 (for BI) or bootstrap values < 70% (for ML). Clades of *Akanthomyces*, *Beauveria*, and *Cordyceps* are highlighted because each of these genera contains more than two species without taxonomic ambiguity

Two phylogeny-building methods (BI & ML) generated identical topologies. Phylogenetic trees constructed by different datasets (DNA & protein), however, showed topological differences (Fig. 6; Additional file 2: Fig. S7). For instance, *Tolypocladium inflatum* grouped together with two other Ophiocordycipitaceae species at a low support (55% ML) in the DNA tree, whereas the three species failed to group together in the protein tree. Besides, fungal species of every family clustered together with high support values (Fig. 6). Phylogenetic relationship among different families also showed subtle differences between DNA and protein trees (Fig. 6; Additional file 2: Fig. S7). Although these variations, *C. blackwelliae* always clustered into the Cordycipitaceae clade, being closely related to *C. chanhua* with a high support (98% in DNA tree and 80% in protein tree).

### Comparison among mitogenomes of five different *Cordyceps* species

**Fig. 7:**
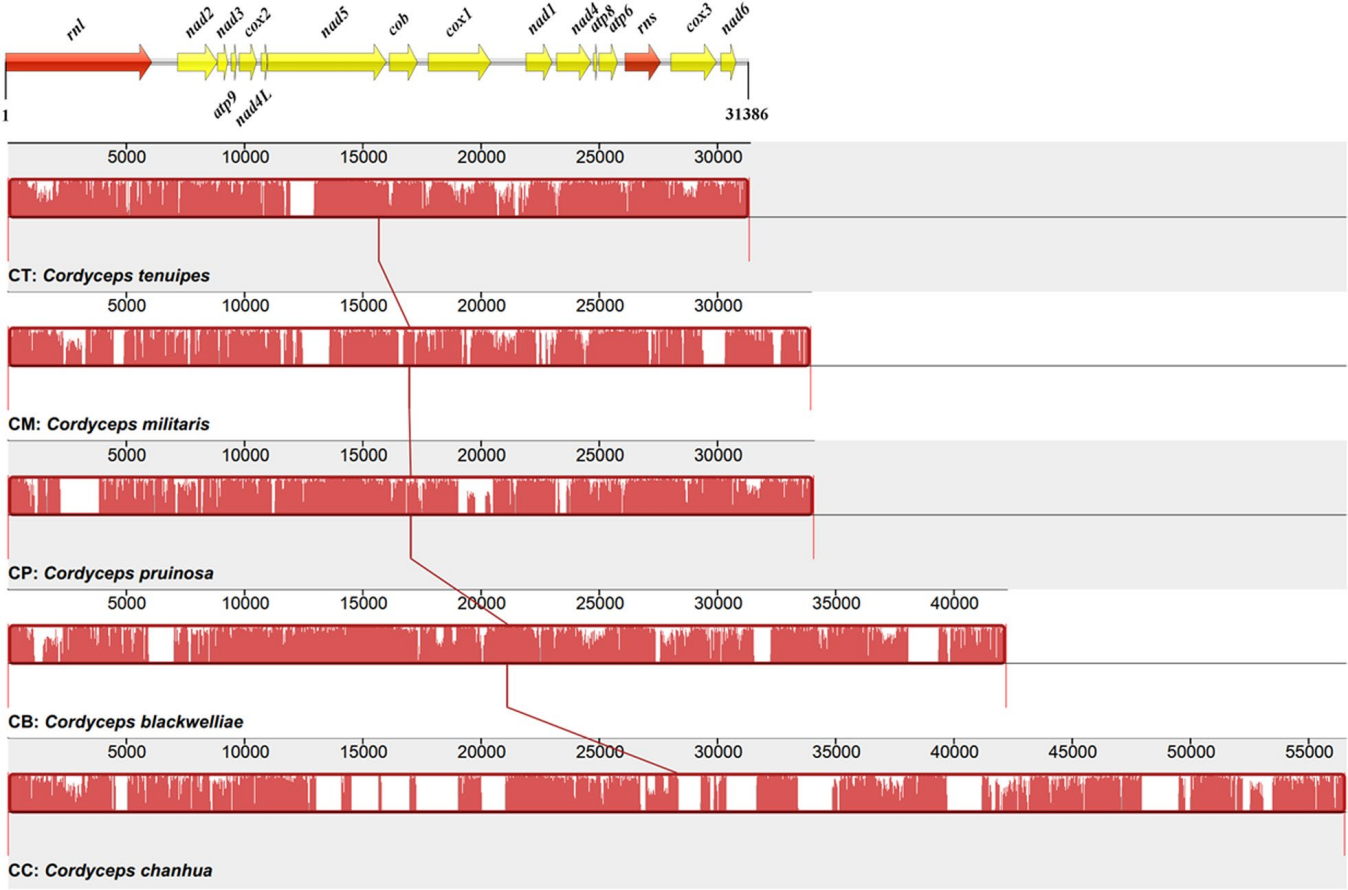
Collinearity among five *Cordyceps* mitogenomes. The progressiveMauve algorithm was used to align these mitogenomes using Mauve. The mitochondrial gene order of *C. tenuipes* is drawn on top of the figure, showcasing the two rRNA genes (in red) and the 14 core PCGs (in yellow)

To have an overall understanding of mitogenome evolution in *Cordyceps*, all five *Cordyceps* species with available mitogenomes (i.e., *C. blackwelliae*, *C. chanhua*, *C. militaris*, *C. pruinosa*, and *C. tenuipes*) were compared. Their mitogenome sizes ranged from 31,386 bp in *C. tenuipes* to 56,581 bp in *C. chanhua* (Table 3). These mitogenomes were consistent in having a low GC content (25.9*–*27.0%), positive AT skew (0.003*–*0.041), and positive GC skew (0.110*–*0.134), indicating higher frequencies of A and G than T and C in the forward strand. As expected, they all contained two rRNA genes and 14 core PCGs, and the arrangement of these genes were conserved among different mitogenomes. This was in concordance with the high synteny among the five mitogenomes (Fig. 7).

**Table 3:** Comparison among mitogenomes of five different *Cordyceps* species

Item	*Cordyceps tenuipes*	*Cordyceps militaris*	*Cordyceps pruinosa*	*Cordyceps blackwelliae*	*Cordyceps chanhua*
Abbreviation	CT	CM	CP	CB	CC
Accession number	MK234910	KP722501	MN515031	OM403992	NC_041489
Strain	YFCC 2017002	V40-5	N/A	ZYJ0835	CCAD02
Mitogenome size (bp)	31,386	33,967	34,094	42,257	56,581
GC content	27.0%	26.8%	26.0%	25.9%	26.1%
AT skew	0.003	0.014	0.009	0.022	0.041
GC skew	0.124	0.134	0.110	0.123	0.119
No. core PCGs	14	14	14	14	14
No. ncORFs	0	1	1	2	3
No. introns	6	8	8	14	25
No. intron-hosting genes	4(*rnl/cox1,3/nad5*)	5(*rnl/cob/cox1-3*)	5(*rnl/cob/cox3/nad1,5*)	7(*rnl/cob/cox1,3/nad1,4,5*)	9(*rnl/atp6,9/cob/cox1-3/nad1,5*)
No. rRNAs	2	2	2	2	2
No. tRNAs	25	27	25	25	25

N/A, not available. ncORFs refer to free-standing ORFs located at intergenic regions of the 14 core PCGs and the two rRNA genes

*Cordyceps* mitogenomes, however, varied in the number of intergenic ORFs (0*–*3), tRNA genes (25*–*27), and introns (6*–*25) (Table 3). Mitogenome size variations were highly correlated with the number of intergenic ORFs (*r* = 0.95) and introns (*r* = 0.99). There was an obviously dynamic distribution of introns among these mitogenomes (Table 4). Specifically, a total of 31 intron insertion sites were present in these five mitogenomes. Almost half of them (14/31) were unique to one species, and the remaining ones were shared by more than two species, leading to a total of 61 introns in these five mitogenomes. Two intron insertions (mL965 and mL2450) in *rnl* were shared by all five species. The mL965 intron contained an ORF encoding a GIY-YIG endonuclease in all species except *C. pruinose*, which lacked an intronic ORF. The mL2450 intron contained an ORF encoding ribosomal protein S3 (i.e., the *rps3* gene) in every species. The ubiquitous nature of the two introns indicates their early invasion before species divergence in *Cordyceps*.

**Table 4:** Intron distribution in different *Cordyceps* mitogenomes

Host gene	Intron	*Cordyceps tenuipes*	*Cordyceps militaris*	*Cordyceps pruinosa*	*Cordyceps blackwelliae*	*Cordyceps chanhua*	Occurrence
*rnl*	mL812		●			●	2
	mL965	●	●	●	●	●	5
	mL1924		●			●	2
	mL1968			●			1
	mL2450	●	●	●	●	●	5
	mL2585				●		1
*atp6*	atp6U572					●	1
*atp9*	atp9P181					●	1
*cob*	cobP393		●	●		●	3
	cobP490			●		●	2
	cobP506				●	●	2
	cobP823					●	1
*cox1*	cox1P212					●	1
	cox1P281				●	●	2
	cox1P709					●	1
	cox1P720				●	●	2
	cox1P731	●	●		●	●	4
	cox1P1057				●	●	2
	cox1P1281					●	1
*cox2*	cox2P228		●			●	2
	cox2P357					●	1
	cox2P651					●	1
*cox3*	cox3P219	●		●	●	●	4
	cox3P333				●		1
	cox3P631		●		●	●	3
*nad1*	nad1P636			●	●	●	3
*nad4*	nad4P505				●		1
*nad5*	nad5P417					●	1
	nad5P570			●		●	2
	nad5P717	●					1
	nad5P924	●			●		2
Sum	31	6	8	8	14	25	61

Solid circles indicate presence of introns

**Fig. 8:**
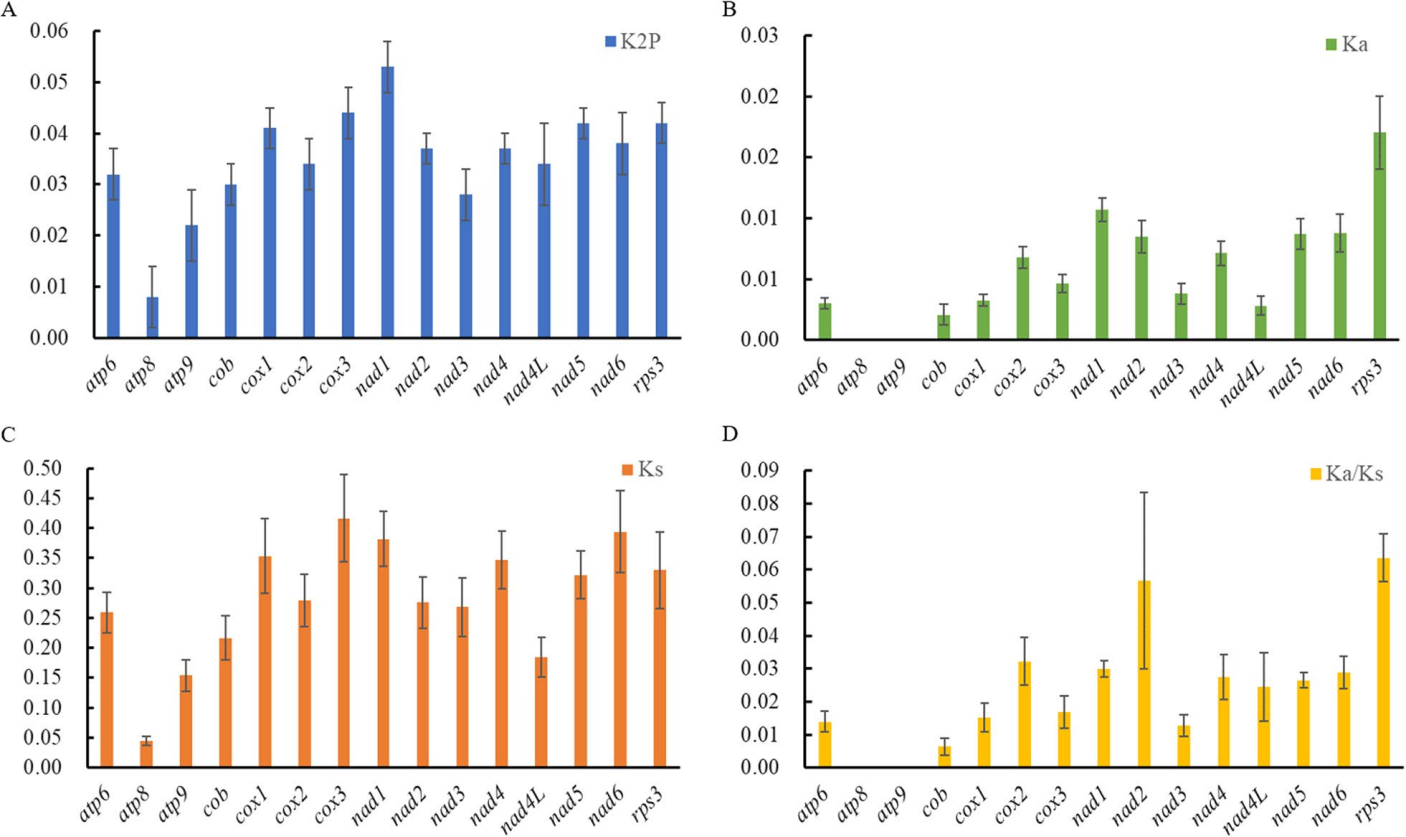
Genetic differentiation of mitochondrial protein coding genes among five *Cordyceps* species. **A** the Kimura-2-parameter (K2P) distance; **B** the number of nonsynonymous substitutions per nonsynonymous site (Ka); **C** the number of synonymous substitutions per synonymous site (Ks); **D** Ka/Ks ratio

We further compared the evolution of 15 PCGs (including 14 core PCGs plus *rps3*) that were present in every mitogenome. Coding sequence of *atp6*, *atp8*, *atp9*, *cox2*, *cox3*, *nad1*, *nad4*, *nad4L* were conserved in length, while *cob*, *cox1*, *nad2*, *nad3*, *nad5*, *nad6*, and *rps3* showed length variations among different species (Additional file 1: Table S5). Of these 15 genes, *nad1* had the largest mean K2P genetic distance, followed by *cox1*, *cox3*, *nad5*, and *rps3* (Fig. 8A; Additional file 1: Tables S5 and S6). The mean K2P genetic distance of *atp8* was the smallest, indicating that this gene is highly conserved across the mitogenomes. The mean Ka value of *rps3* was the highest among all PCGs, while that of *atp8* and *atp9* were the lowest (Fig. 8B). The mean Ks values of *cox3*, *nad1*, and *nad6* were higher than other PCGs (Fig. 8C). The highest Ka/Ks ratios were found for *rps3* and *nad2*, and Ka/Ks ratios for all genes were lower than 1 (Fig. 8D; Additional file 1: Tables S5 and S6), suggesting that all genes evolved under purifying selection.

## DISCUSSION

The study tested whether different *Cordyceps* species are conserved in their mitogenome organization and if alternative splicing event exists for intron-containing mitochondrial genes in filamentous fungi. Comparison of mitogenomes among species of *Cordyceps* revealed conservation in mitogenome organization although differentiation has occurred since a most recent common ancestor. Alternative splicing events were obvious for several intron-containing mitochondrial genes in *C. blackwelliae*.

This study reported the mitogenome of *C. blackwelliae* for the first time. Its mitogenome size at 42.3 kb is smaller than most Hypocreales species (average at 52.1 kb, median at 46.9 kb) but larger than most Cordycipitaceae species (average at 33.2 kb, median at 29.0 kb) (Additional file 2: Fig. S8). Within Cordycipitaceae, the mitogenome size of *C. blackwelliae* is smaller than that of three species, namely *Beauveria lii* (59.0 bp, MT818175), *Beauveria malawiensis* (44.1 kb, NC_030635), and *Cordyceps chanhua* (56.6 kb, NC_041489), and larger than that of other 13 species by referring to data in Supplementary Table S5 in Ren et al. 2021. The mitogenome expansion of *C. blackwelliae* is attributed to intron insertion events. A total of 14 introns (18.2 kb, 43.0% of the mitogenome) were identified from seven genes in the *C. blackwelliae* mitogenome. Mitogenome size variations have been shown to be highly correlated with the number of introns in many fungal lineages (Fan et al. 2019; Zhang et al. 2019, 2022). In addition, as observed in all other Cordycipitaceae species (Ren et al. 2021), genes *nad2* and *nad3* overlap by one base in *C. blackwelliae.* Besides the 1-bp overlapping, there can be 0, 1, 4, or 6-bp separation between *nad2* and *nad3* in different species in Hypocreales (Ren et al. 2021). Genes *nad4L* and *nad5* overlap by one base in all Hypocreales species with available mitogenomes (Ren et al. 2021).

Annotation of the *C. blackwelliae* mitogenome, especially our prediction of introns, was supported by RNA-Seq results. We found that different pre-treatment strategies of RNA samples influenced the amount of sequencing reads representing mitochondrial RNAs, with the RD strategy retaining more mitochondrial reads than the PA strategy. This indicates that, unlike nuclear mRNAs, mitochondrial mRNAs may lack typical polyA tails in their mature transcripts.

According to results of the RD strategy, strong expression was confirmed for most mitochondrial genes but with clear expression differences among different genes. For example, *atp8* and *atp6* of *C. blackwelliae* located adjacently in the mitogenome (with 80-bp separation) (Table 1), and they transcribed as the same polycistronic transcript (Fig. 4B). Nevertheless, there was a high FPKM value for *atp6* but zero for *atp8* (Additional file 1: Table S2). Lower expression of *atp8* than *atp6* was also documented in other fungi, such as *Ophiocordyceps sinensis* (Li et al. 2015), *Pestalotiopsis fici* (Zhang et al. 2017a), *Tolypocladium inflatum* (Zhang et al. 2017b), and *Hirsutella thompsonii* (Wang et al. 2018). Since mitochondrial genes are transcribed as polycistronic transcripts, expression difference among mitochondrial genes is most likely due to their differences in mitochondrial RNA stability (Shang et al. 2018). In this study, only few reads mapped to *trn* genes of *C. blackwelliae* (Additional file 2: Fig. S4C), whereas strong expression of *trn* genes was reported in some other fungi (Kolondra et al. 2015; Shang et al. 2018; Turk et al. 2013). This difference may be due to the fact that only fragments of 350–400 bp were extracted and used for sequencing in our study.

For non-conserved genes (i.e., intronic and intergenic ORFs) of *C. blackwelliae*, their active transcription was also detected according to both FPKM values and local BLASTN searches (Fig. 3; Additional file 1: Tables S2 and S3). Similar results was also reported in other fungi such as *Ophiocordyceps sinensis* (Li et al. 2015). These non-conserved genes display important functions. For example, intron encoded proteins LAGLIDADG or GIY-YIG endonucleases function in intron mobility (Mukhopadhyay and Hausner 2021). Ribosomal protein S3 contributes to ribosome assembly (Korovesi et al. 2018).

Similar to those reported in other fungi (Kolondra et al. 2015; Schäfer et al. 2005; Turk et al. 2013), transcription as polycistronic units was observed for mitochondrial genes of *C. blackwelliae*. It is also possible for mitochondrial genes of *C. blackwelliae* to transcribe into one long polycistronic transcript (Fig. 4; Additional file 2: Fig. S5). Promoters and origin of replication remain to be determined. Evidence of alternative splicing was detected for three (i.e., *cob*, *cox1*, and *nad5*) of the seven intron-containing genes in *C. blackwelliae* (Fig. 5). Mechanism of mitochondrial alternative splicing remains to be determined.

Phylogenetic analyses were performed based on mitochondrial DNA and protein sequences. These analyses included eight families of Hypocreales, namely Bionectriaceae, Clavicipitaceae, Cordycipitaceae, Hypocreaceae, Nectriaceae, Ophiocordycipitaceae, Sarocladiaceae, and Stachybotryaceae. These are the families with available mitogenome information in Hypocreales to date. There are currently 14 accepted families in Hypocreales (Hyde et al. 2020), and other six families (i.e., Calcarisporiaceae, Cocoonihabitaceae, Flammocladiellaceae, Myrotheciomycetaceae, Niessliaceae, and Tilachlidiaceae) still lack available mitogenomes. As expected, *C. blackwelliae* clustered into the Cordycipitaceae clade (Fig. 6; Additional file 2: Fig. S7). Cordycipitaceae species with available mitogenomes are from the following seven genera: *Akanthomyces*, *Beauveria*, *Cordyceps*, *Isaria*, *Lecanicillium*, *Parengyodontium*, and *Samsoniella*. For three of these genera, *Akanthomyces*, *Beauveria*, and *Cordyceps*, each contained more than two species in our study, and fungal taxa from each of these genera grouped together (Fig. 6). Two *Lecanicillium* species (*Lecanicillium saksenae* and *Lecanicillium fungicola*) were also employed in this study, but they did not cluster together (Fig. 6). This is consistent to the polyphyly of *Lecanicillium* and the suggested suppression of this generic name in Cordycipitaceae (Kepler et al. 2017). Actually, several species previously placed in *Lecanicillium* have been transferred into other genera, including *Akanthomyces*, *Flavocillium*, and *Gamszarea* (Kepler et al. 2017; Wang et al. 2020; Zhang et al. 2020). *Akanthomyces lecanii* and *Akanthomyces muscarius* used in this study were previously placed in *Lecanicillium* (Kepler et al. 2017). Species presently placed in *Lecanicillium* remain to be investigated taxonomically, including the two used in this study. Taxonomy of the strain ARSEF 3 currently under the name *Isaria farinosa* also needs to be clarified because *Isaria farinosa* has been renamed as *Cordyceps farinosa* based on phylogenetic position of the ex-epitype isolate CBS 111113 (Kepler et al. 2017). ARSEF 3 seems to be phylogenetically distant from CBS 111113 (Zhang and Zhang 2019b), and is likely to cluster into the genus *Samsoniella* (Unpublished data). The family Cordycipitaceae currently contains 20 accepted generic names, not including *Isaria* and *Lecanicillium* (Hyde et al. 2020; Wang et al. 2020; Zhang et al. 2020)*.* Sequencing mitogenomes of representative species from other genera in Cordycipitaceae is of great urgency in order to fully understand the phylogeny and evolution in Cordycipitaceae.

*Cordyceps blackwelliae* represents the fifth species with available mitogenome in *Cordyceps*. Comparison among mitogenomes of the five *Cordyceps* species was further performed. They displayed conserved gene order regarding rRNA and typical PCGs, but different numbers of intergenic ORFs and introns contributed to their mitogenome size variations. Different PCGs showed variable genetic diversity, but they were all under purifying selection. *Cordyceps* is a genus of about 180 described species (https://nmdc.cn/fungalnames/). Mitogenomes of additional *Cordyceps* species also need to be sequenced in order to have an overall understanding of mitogenome evolution in *Cordyceps*.

## CONCLUSIONS

This study reported the mitogenome of *Cordyceps blackwelliae*. The exon–intron structure inferred by in silico approach was supported by RNA-Seq experiments. We also identified differential expression, polycistronic transcription units, and alternative splicing for mitochondrial genes in the fungus. Comparison among mitogenomes of different *Cordyceps* species revealed conservation in overall gene arrangement as well as variabilities in intron insertion and genetic differentiation of different genes. Phylogenetic analysis supported its taxonomic ranking within Cordycipitaceae. This study contributes to understanding mitogenome evolution in *Cordyceps*.

## Supplementary Information

**Additional file 1**. **Table S1** Species information used for constructing phylogenetic trees. **Table S2** FPKM values of mitochondrial genes from two different RNA-Seq strategies. **Table S3** Local BLAST of mitochondrial genesagainst assembled transcript contigs. **Table S4** Local BLAST of mitochondrial intron-containing genesagainst assembled transcript contigs. **Table S5** Length, mean K2P genetic distances and Ka/Ks ratios of protein-coding gene among five different *Cordyceps* species. **Table S6** K2P genetic distances and Ka/Ks ratios between different species pairs at protein-coding genes.

**Additional file 2**. **Fig. S1** Comparison on sequencing depth between mtDNA and nuclear DNA. **Fig. S2** Secondary structure of tRNA genes encoded in the *Cordyceps blackwelliae* mitogenome. **Fig. S3** Visualization of transcriptome reads mapping to the *C. blackwelliae* mitogenome using IGV. **Fig. S4** Sequencing depth at *atp8*, *orf112*, and *rnl/nad2* intergenic *trn* genesfrom rRNA-depletion strategy RNA-Seq. **Fig. S5** PCR assays of five fragments. **Fig. S6** BLASTN analysis of the mitogenome against 44 de novo assembled transcripts. **Fig. S7** Phylogenetic analysis of Hypocreales species based on concatenated protein sequences of 14 typical mitochondrial PCGs. **Fig. S8** Distribution of mitogenome sizes for fungi in Hypocreales and Cordycipitaceae.

## Data Availability

All data used in this study are publicly available.

## Abbreviations

Mitogenome: Mitochondrial genome
BI: Bayesian inference
ML: Maximum likelihood
PCG: Protein-coding gene
Ks: Synonymous substitution rate
Ka: Nonsynonymous substitution rate
PA: PolyA RNA capture strategy for RNA-Seq
RD: Ribosomal RNA depletion strategy for RNA-Seq
