# Supplementary material for: Mitochondrial genome of Cordyceps blackwelliae: organization, transcription, and evolutionary insights into Cordyceps
Source: IMA Fungus. 2023 Jul 6;14:13. doi: 10.1186/s43008-023-00118-5 (PMC10327131; doi:10.1186/s43008-023-00118-5)
Supplement: Supplementary file 2 — Additional file 2. Fig. S1 Comparison on sequencing depth between mtDNA and nuclear DNA. Fig. S2 Secondary structure of tRNA genes encoded in the Cordyceps blackwelliae mitogenome. Fig. S3 Visualization of transcriptome reads mapping to the C. blackwelliae mitogenome using IGV. Fig. S4 Sequencing depth at atp8, orf112, and rnl/nad2 intergenic trn genesfrom rRNA-depletion strategy RNA-Seq. Fig. S5 PCR assays of five fragments. Fig. S6 BLASTN analysis of the mitogenome against 44 de novo assembled transcripts. Fig. S7 Phylogenetic analysis of Hypocreales species based on concatenated protein sequences of 14 typical mitochondrial PCGs. Fig. S8 Distribution of mitogenome sizes for fungi in Hypocreales and Cordycipitaceae. [file 43008_2023_118_MOESM2_ESM.pptx]

## Slide 1
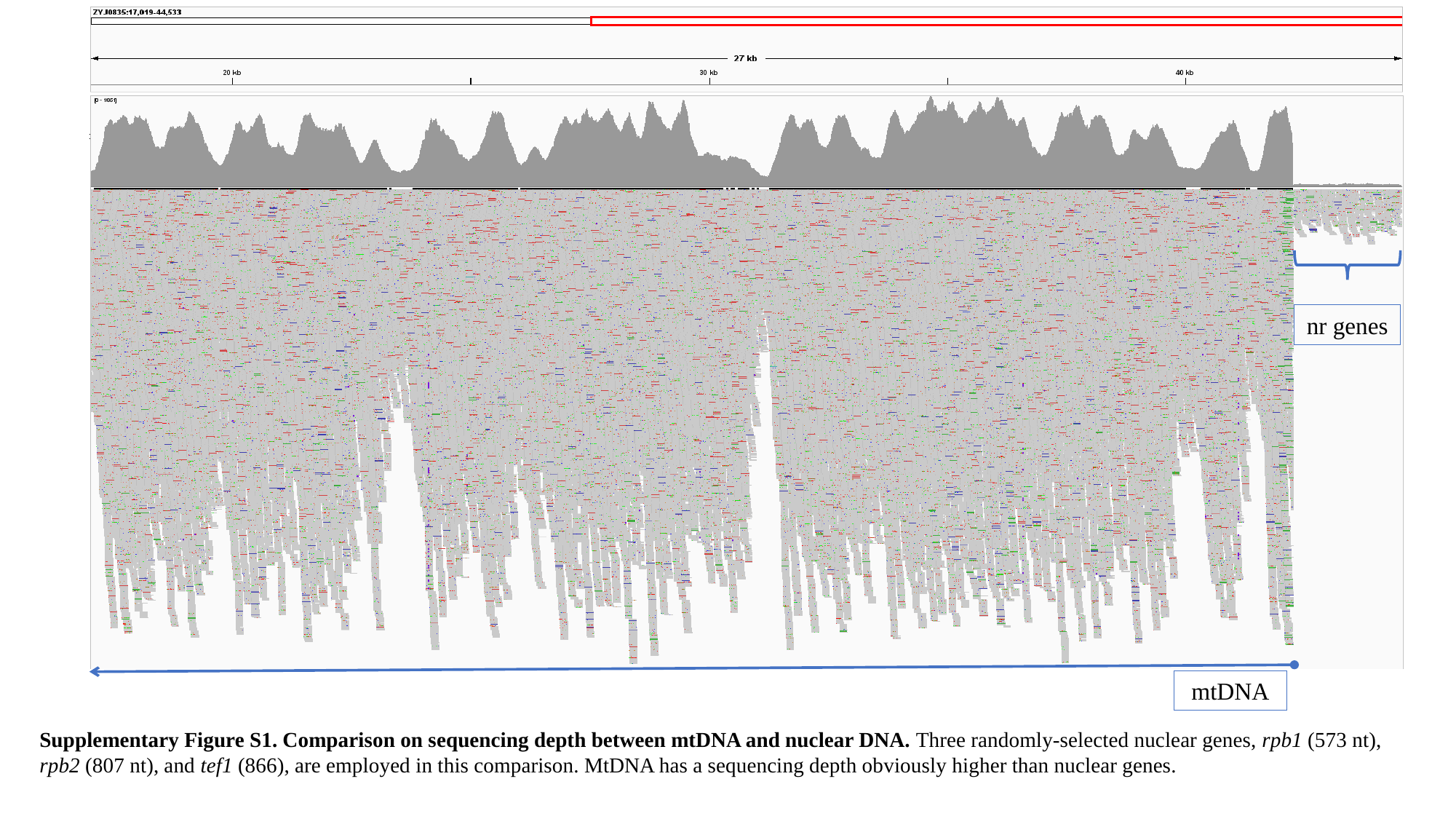

nr genes
mtDNA
Supplementary Figure S1. Comparison on sequencing depth between mtDNA and nuclear DNA. Three randomly-selected nuclear genes, rpb1 (573 nt), rpb2 (807 nt), and tef1 (866), are employed in this comparison. MtDNA has a sequencing depth obviously higher than nuclear genes.

## Slide 2
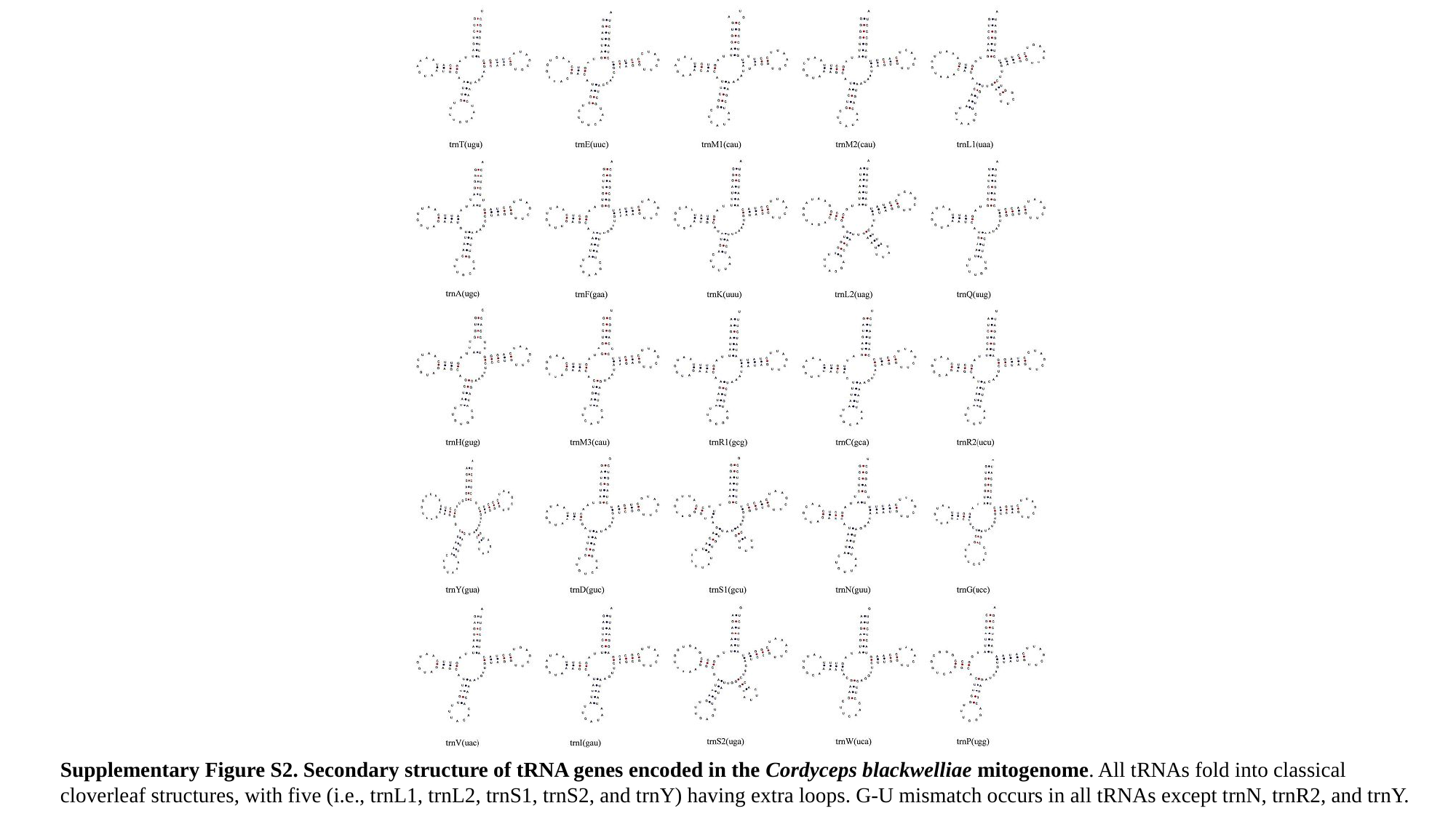

Supplementary Figure S2. Secondary structure of tRNA genes encoded in the Cordyceps blackwelliae mitogenome. All tRNAs fold into classical cloverleaf structures, with five (i.e., trnL1, trnL2, trnS1, trnS2, and trnY) having extra loops. G-U mismatch occurs in all tRNAs except trnN, trnR2, and trnY.

## Slide 3
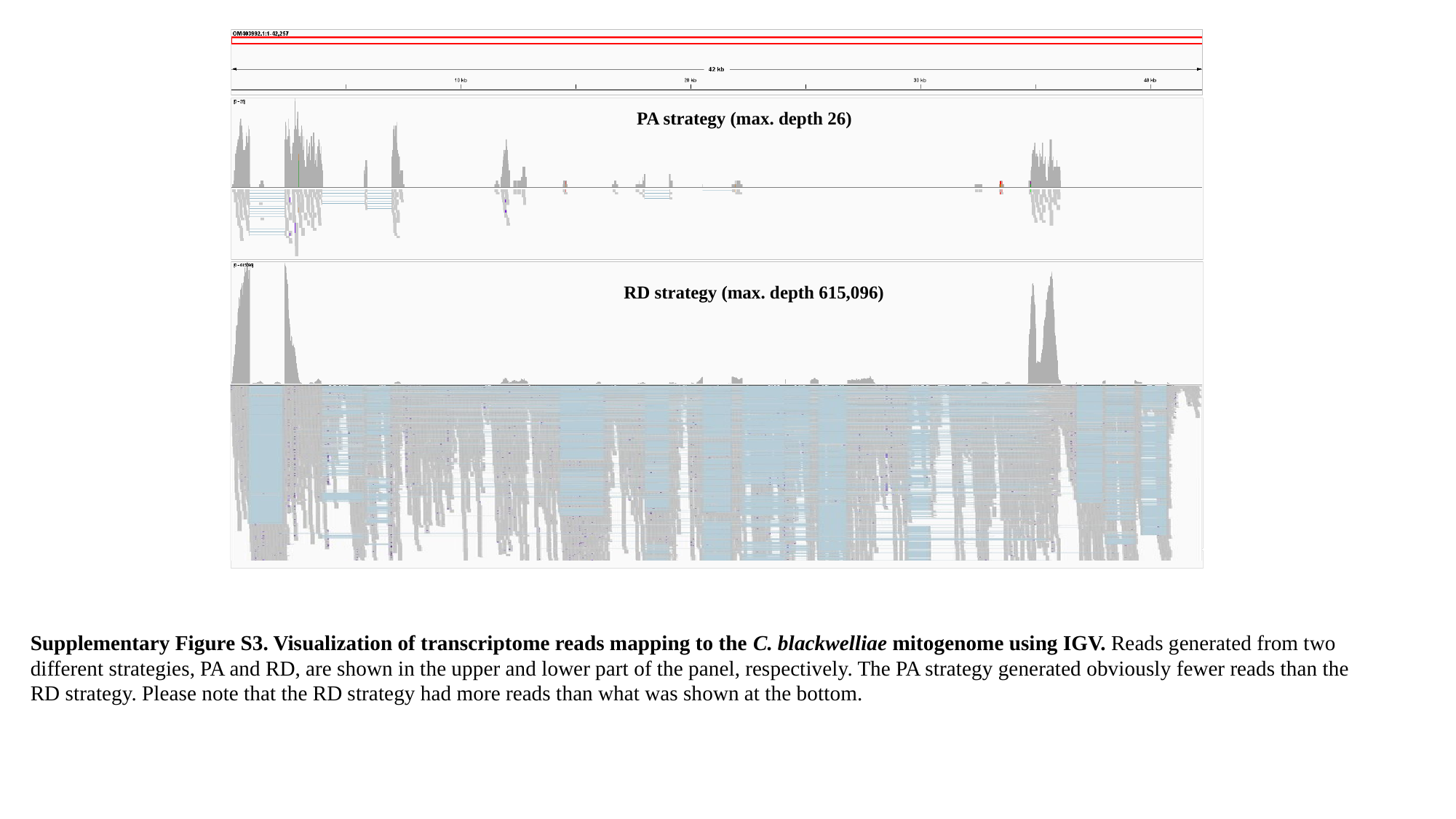

PA strategy (max. depth 26)
RD strategy (max. depth 615,096)
Supplementary Figure S3. Visualization of transcriptome reads mapping to the C. blackwelliae mitogenome using IGV. Reads generated from two different strategies, PA and RD, are shown in the upper and lower part of the panel, respectively. The PA strategy generated obviously fewer reads than the RD strategy. Please note that the RD strategy had more reads than what was shown at the bottom.

## Slide 4
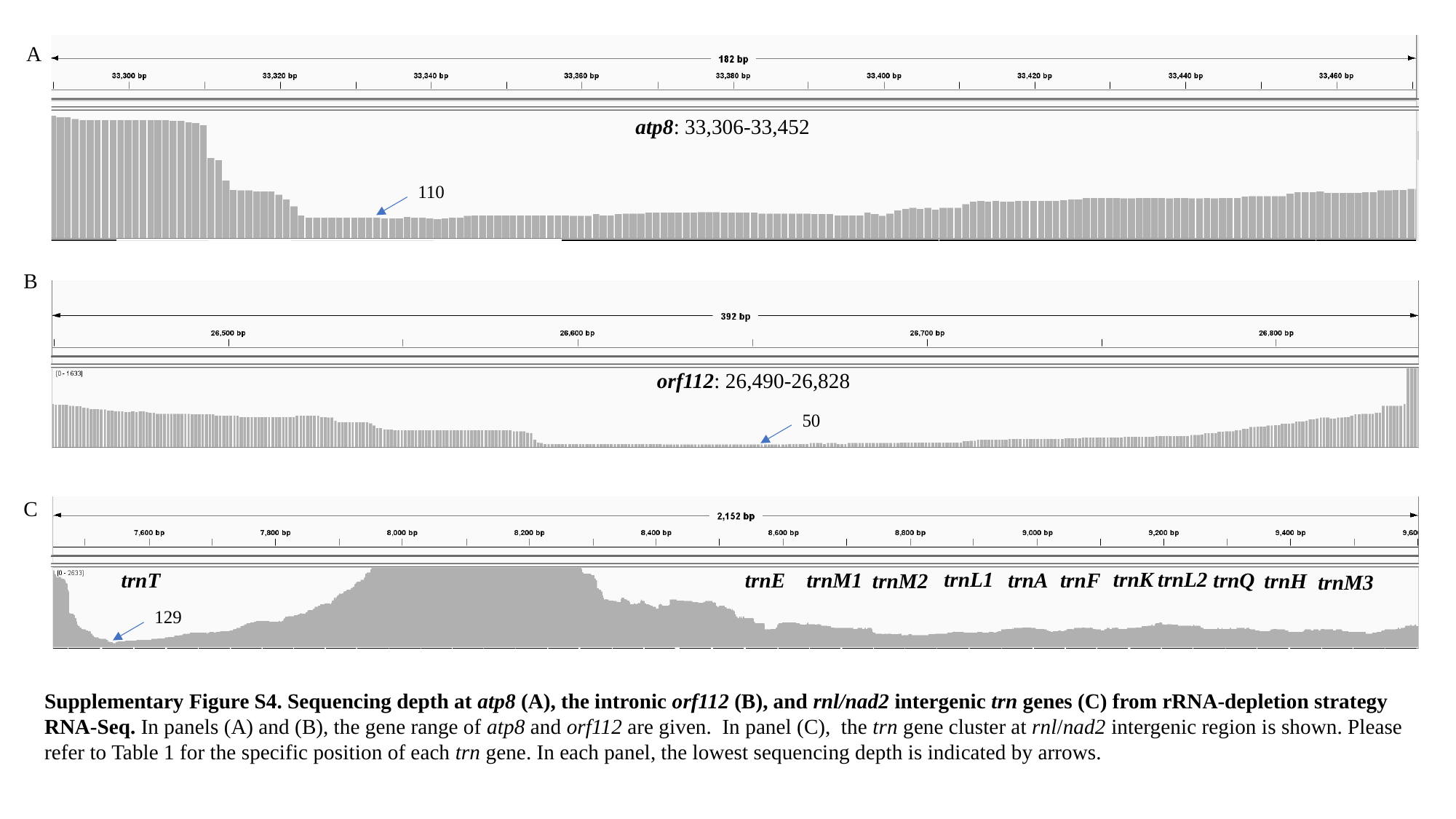

A
atp8: 33,306-33,452
110
B
orf112: 26,490-26,828
50
C
trnL1
trnL2
trnK
trnQ
trnT
trnF
trnE
trnM1
trnA
trnM2
trnH
trnM3
129
Supplementary Figure S4. Sequencing depth at atp8 (A), the intronic orf112 (B), and rnl/nad2 intergenic trn genes (C) from rRNA-depletion strategy RNA-Seq. In panels (A) and (B), the gene range of atp8 and orf112 are given. In panel (C), the trn gene cluster at rnl/nad2 intergenic region is shown. Please refer to Table 1 for the specific position of each trn gene. In each panel, the lowest sequencing depth is indicated by arrows.

## Slide 5
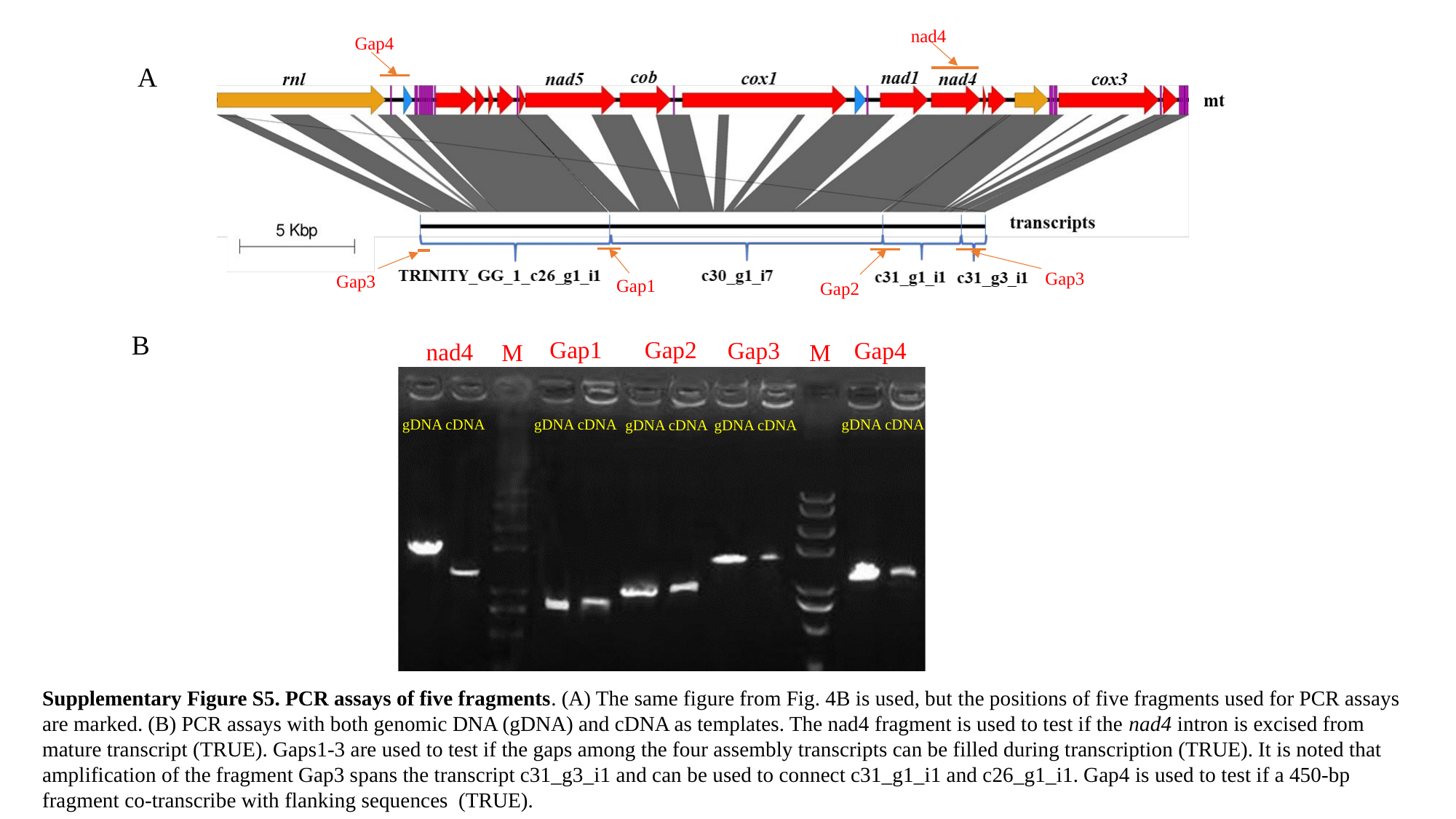

nad4
Gap4
A
Gap3
Gap3
Gap1
Gap2
B
Gap1
Gap2
Gap4
Gap3
nad4
M
M
gDNA cDNA
gDNA cDNA
gDNA cDNA
gDNA cDNA
gDNA cDNA
Supplementary Figure S5. PCR assays of five fragments. (A) The same figure from Fig. 4B is used, but the positions of five fragments used for PCR assays are marked. (B) PCR assays with both genomic DNA (gDNA) and cDNA as templates. The nad4 fragment is used to test if the nad4 intron is excised from mature transcript (TRUE). Gaps1-3 are used to test if the gaps among the four assembly transcripts can be filled during transcription (TRUE). It is noted that amplification of the fragment Gap3 spans the transcript c31_g3_i1 and can be used to connect c31_g1_i1 and c26_g1_i1. Gap4 is used to test if a 450-bp fragment co-transcribe with flanking sequences (TRUE).

## Slide 6
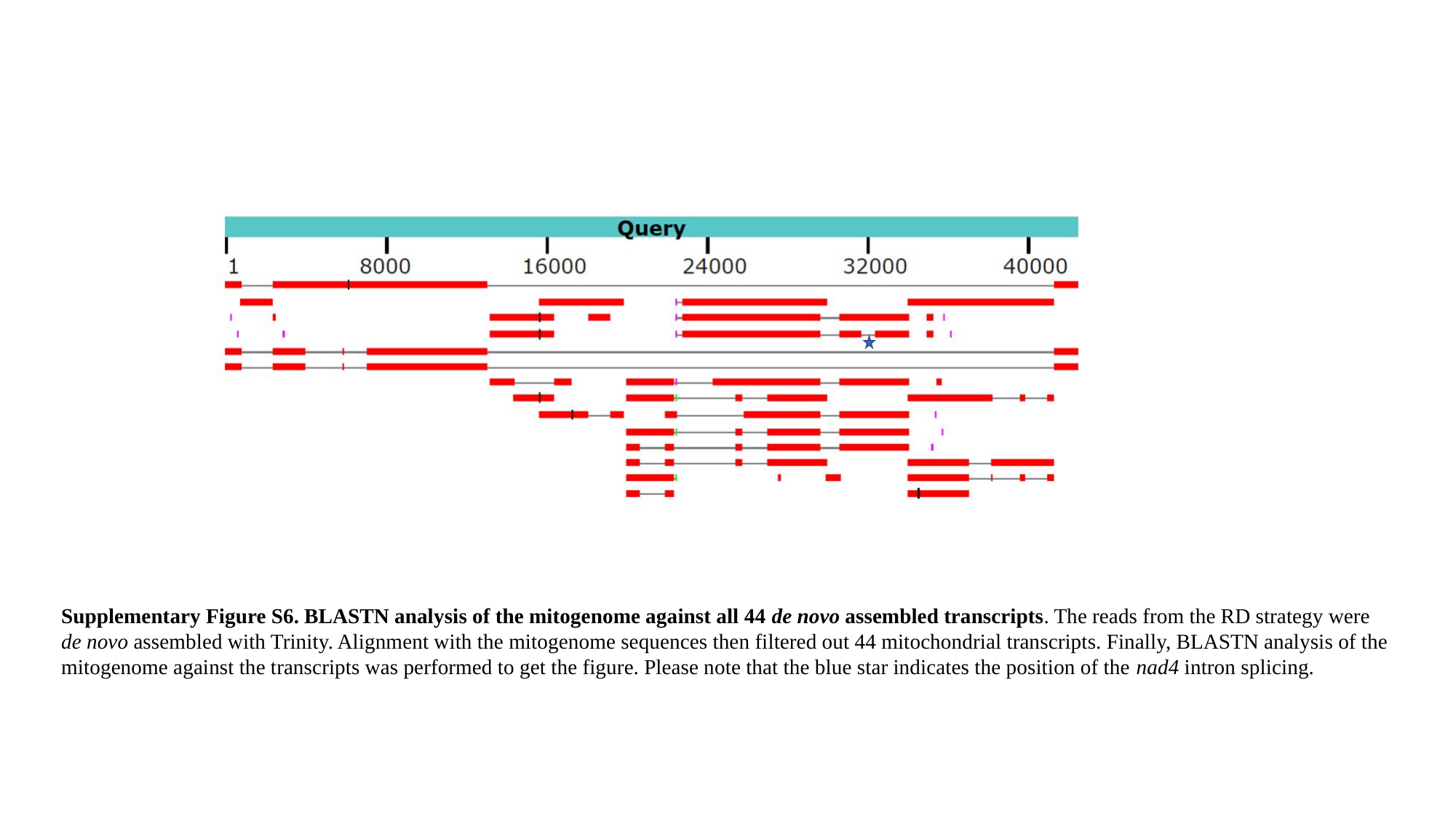

Supplementary Figure S6. BLASTN analysis of the mitogenome against all 44 de novo assembled transcripts. The reads from the RD strategy were de novo assembled with Trinity. Alignment with the mitogenome sequences then filtered out 44 mitochondrial transcripts. Finally, BLASTN analysis of the mitogenome against the transcripts was performed to get the figure. Please note that the blue star indicates the position of the nad4 intron splicing.

## Slide 7
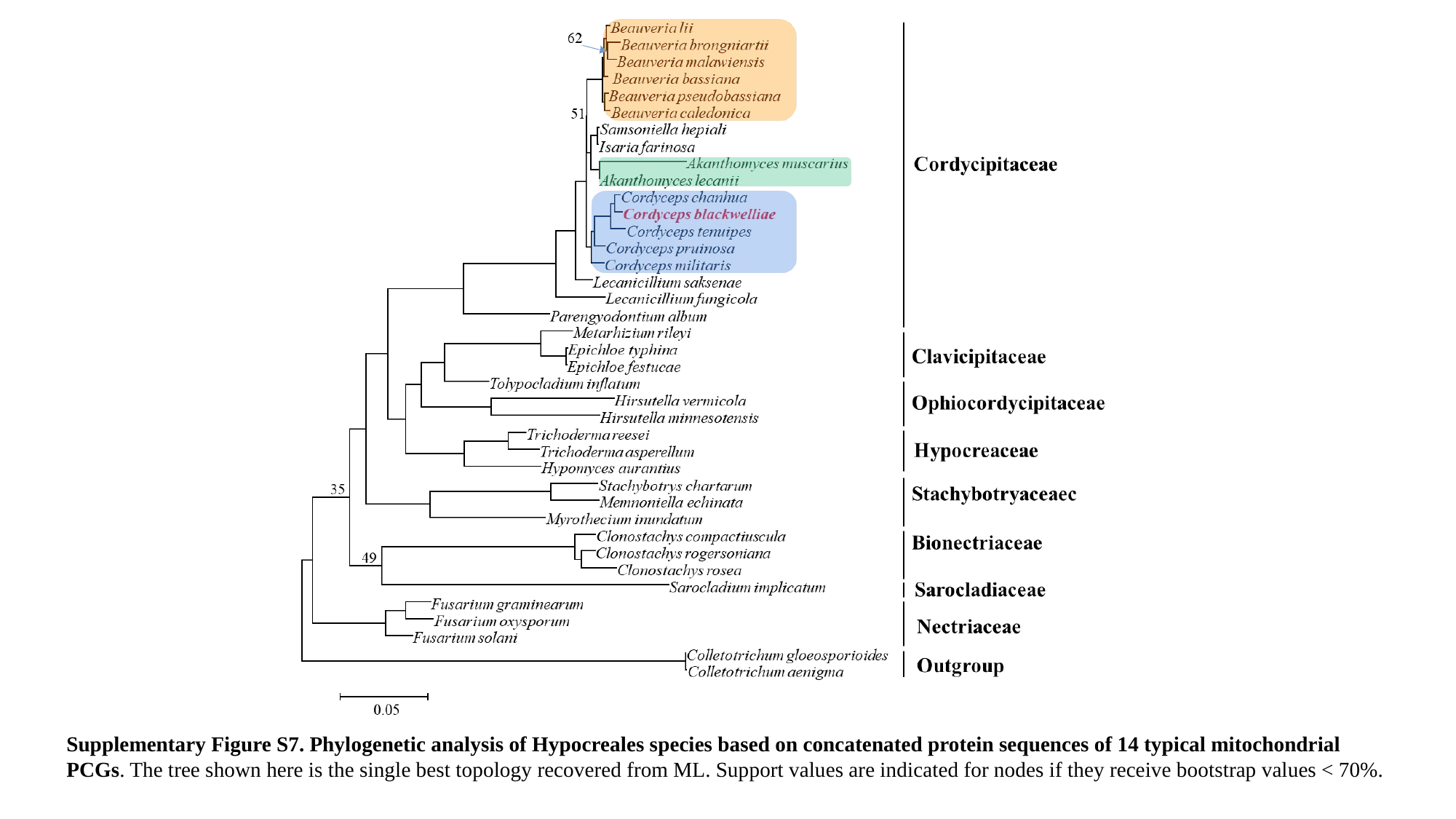

Supplementary Figure S7. Phylogenetic analysis of Hypocreales species based on concatenated protein sequences of 14 typical mitochondrial PCGs. The tree shown here is the single best topology recovered from ML. Support values are indicated for nodes if they receive bootstrap values < 70%.

## Slide 8
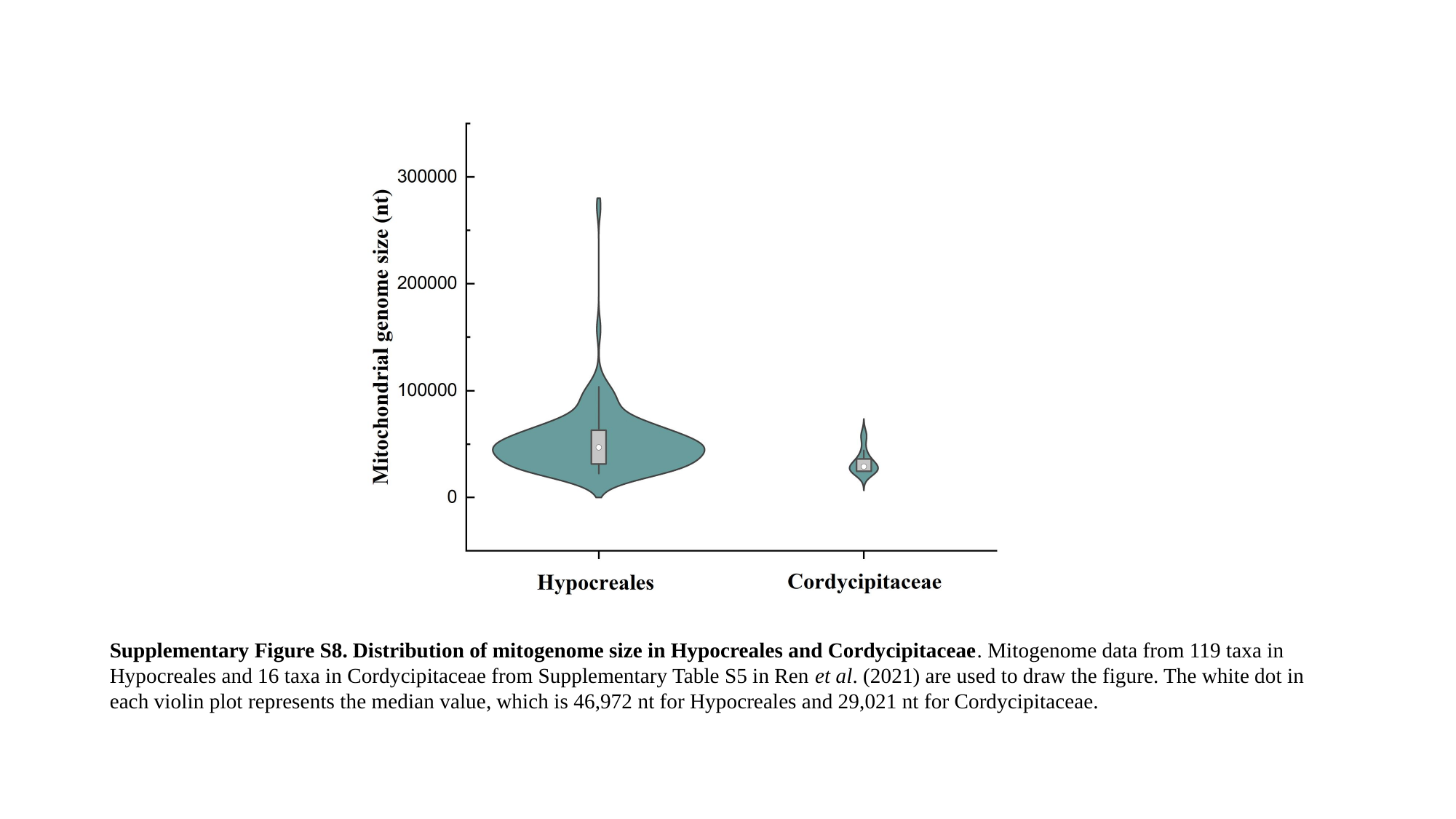

Supplementary Figure S8. Distribution of mitogenome size in Hypocreales and Cordycipitaceae. Mitogenome data from 119 taxa in Hypocreales and 16 taxa in Cordycipitaceae from Supplementary Table S5 in Ren et al. (2021) are used to draw the figure. The white dot in each violin plot represents the median value, which is 46,972 nt for Hypocreales and 29,021 nt for Cordycipitaceae.
